# Bacterial Signal Peptides- Navigating the Journey of Proteins

**DOI:** 10.3389/fphys.2022.933153

**Published:** 2022-07-26

**Authors:** Sharbani Kaushik, Haoze He, Ross E. Dalbey

**Affiliations:** Department of Chemistry and Biochemistry, The Ohio State University, Columbus, OH, United States

**Keywords:** SecYEG translocase, SecA, signal peptide, protein transport, YidC, Tat pathway, signal peptidase, antibiotic targets

## Abstract

In 1971, Blobel proposed the first statement of the Signal Hypothesis which suggested that proteins have amino-terminal sequences that dictate their export and localization in the cell. A cytosolic binding factor was predicted, and later the protein conducting channel was discovered that was proposed in 1975 to align with the large ribosomal tunnel. The 1975 Signal Hypothesis also predicted that proteins targeted to different intracellular membranes would possess distinct signals and integral membrane proteins contained uncleaved signal sequences which initiate translocation of the polypeptide chain. This review summarizes the central role that the signal peptides play as address codes for proteins, their decisive role as targeting factors for delivery to the membrane and their function to activate the translocation machinery for export and membrane protein insertion. After shedding light on the navigation of proteins, the importance of removal of signal peptide and their degradation are addressed. Furthermore, the emerging work on signal peptidases as novel targets for antibiotic development is described.

## 1 Introduction

The transport of proteins across cell membranes is fundamentally significant to many biological processes. Protein export also finds a special interest in biotechnology for production of hormones/enzymes and recombinant proteins, in laboratory techniques and disease diagnosis. Considerable progress has been made during the last several decades in understanding the characteristics of the folded state of substrates during translocation in the cytosol, membrane targeting, the structure and function of translocation devices, the insertion of membrane proteins into the lipid bilayer, and the role of energy in protein export. Insight into these fundamental concepts is highly appreciated and anticipated by scientists in the protein export field.

Protein integration and transport across the membranes are ubiquitous in every organism. Typically, these proteins are synthesized with a stretch of amino acids called the “signal peptide” that can be recognized by the cytosolic proteins for sorting and then targeting to the membrane. After being transferred to the translocation machinery, the proteins are membrane inserted or translocated across the membrane. In the final step, the signal peptide is proteolytically removed from the exported protein by signal peptidase.

The signal peptide plays center stage in this export process with a myriad of functions (Hegde and Bernstein, 2006). The signal peptides can bind to chaperones to prevent premature folding of the protein in the cytosol. In addition to slowing down the folding of a mature domain of a preprotein, signal peptides act as a zip code for sorting the proteins from the cytosol to the membrane. Finally, the signal peptide activates the translocation machinery, initiating the translocation process.

This review highlights the function of signal peptides in Gram-negative bacteria in protein sorting and targeting to the inner membrane, and translocation across the membrane and insertion. After navigating the journey of proteins, their removal and degradation are discussed. Furthermore, the potential of the signal peptidases (endopeptidases which remove signal peptides) as antibacterial targets will be covered.

## 2 Signal Peptides

Most exported proteins in bacteria are transported across the inner membrane by the general secretion (Sec) pathway or the Twin arginine translocation (Tat) system or the simple membrane protein insertase YidC [reviewed in (Crane and Randall, 2017; Frain et al., 2019; Shanmugam and Dalbey, 2019; Oswald et al., 2021)]. The targeting of the preproteins to these pathways are dependent on the pathway selective for the respective signal peptide. These are the Sec signal peptide, the lipoprotein signal peptide, the Tat signal peptide and the prepilin signal peptide. Below we describe the properties of each of these signal peptides.

**FIGURE 1:**
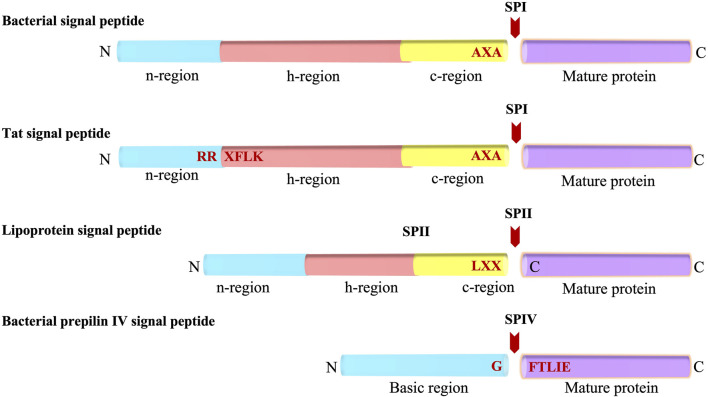
Bacterial Signal peptides. Schematic representations of the Sec-type signal peptide, the twin-arginine (Tat) signal peptide, the lipoprotein signal peptide, and the prepilin signal peptide. The various regions of the signal peptides (n, h, c and basic regions) are indicated. The SP cleavage site is represented with a red arrow. N and C indicates amino and carboxyl-terminus, respectively.

The Sec signal peptide targets the protein to the Sec machinery and is composed of three regions (Figure 1) (von Heijne and Abrahmsèn, 1989; Perlman and Halvorson, 1983; von Heijne, 1986a): 1) a positively charged N-terminal region (n), 2) a central hydrophobic region (h) and, 3) a rather polar C-terminal region which contains small amino acid residues at positions -1 and -3 (with respect to the cleavage site). Additionally, a helix breaking residue is often found at the -4 to -6 positions of the C-terminal region (von Heijne, 1986a). Genetic and mutagenesis studies have shown that the apolar region of the signal peptide is essential for the function of a cleavable signal peptide (Emr et al., 1978; Bassford and Beckwith, 1979; Michaelis and Beckwith, 1982). Moreover, the basic amino terminus can be important for making translocation more efficient (Vlasuk et al., 1983; Iino et al., 1987).

Similar to the Sec signal peptide, the lipoprotein signal peptide which is processed by signal peptidase 2 (SPase II, lipoprotein signal peptidase) has a positively charged n region and a hydrophobic central region (h region) (Figure 1). The main difference between Sec and lipoprotein signal sequences is that the c region of the lipoprotein contains the lipobox motif comprised of Leu-Ala/Ser-Gly/Ala-Cysteine at the −3 to +1 position (Sankaran and Wu, 1994). The lipobox motif is a structural determinant for lipid modification of the strictly conserved Cys at the +1 position of the mature domain that gets modified by diacylglyceride. The glyceride fatty acid lipid is attached by a preprolipoprotein diacylglycerol transferase (Lgt), prior to cleavage by SPase II (Sankaran and Wu, 1994). In Gram-negative and some Gram-positive bacteria, most lipoproteins are further modified by an acyl chain by N-acyl transferase (Lnt) after SPase II cleavage (Gupta and Wu, 1991). Analysis of the signal sequences have shown that the h regions are shorter for lipoprotein signal peptides as compared to that present in the Sec signal peptides (Klein et al., 1988; von Heijne, 1989; Tjalsma et al., 2000).

The Tat signal peptide targets proteins to the Tat machinery and has a tripartite arrangement similar to the Sec signal peptide (Figure 1). It was initially discovered in chloroplast in exported proteins transported into the thylakoid lumen independently of ATP hydrolysis. Later, Berks and others observed it in cofactor containing periplasmic proteins of bacteria (Chaddock et al., 1995; Berks, 1996; Bogsch et al., 1998; Sargent et al., 1998; Weiner et al., 1998). The “Tat” signal peptide takes its name from the invariant and essential twin arginines in the n-region of the signal peptide. The motif for Tat signal peptides is RRXFLK where X can be any residue and F, L and K are quite commonly found. Mutagenesis of the twin arginines even to a lysine pair can abolish or significantly reduce transport although single mutations of the arginines are largely tolerated (Stanley et al., 2000; Buchanan et al., 2001; DeLisa et al., 2002). Typically, the Tat signal peptides are longer than the Sec signal peptides, and the h-region is less hydrophobic than that present in the Sec signal peptides (Cristobal et al., 1999). Moreover, there is often a basic residue in the c-region that functions as a Sec avoidance sequence (Bogsch et al., 1997). While most of the Tat preproteins are processed by signal peptidase 1 (SPase I) (Lüke et al., 2009), some contain a lipobox and are therefore processed by SPase II.

A specialized signal peptide called the prepilin signal peptide is found on the type 4 pilus proteins. Similar to the Sec and most lipoprotein signal peptides, it targets the protein to the Sec machinery. Type 4 substrates are found on the surface of many Gram-negative bacteria such as *Pseudomonas aeruginosa* and *Neisseria gonorrhoeae*. Pilin subunits allow the bacteria to stick to the surface of the host epithelial cells during infection. The prepilin signal peptide is unique as it is cleaved at the border of the n-h region (Strom and Lory, 1993; Mattick, 2002). The processing is carried out by prepilin signal peptidase, which recognizes the GFTLIE motif and cleaves after the glycine (Nunn and Lory, 1991). After cleavage, the prepilin signal peptidase methylates the amino terminus of the mature pilin (Strom et al., 1993). This generates N-methylphenylalanine as the first amino acid of the mature pilin.

In addition to these cleavable signal peptides, uncleaved signal peptides containing a longer hydrophobic stretch target proteins to the translocation machinery but remain as a membrane anchor sequence. These uncleavable signal peptides are found in membrane proteins which span the bacterial inner membrane as an α-helix. These domains are enriched in hydrophobic residues such as Ala, Ile, Leu, and Val but mostly void of charged residues (von Heijne, 2006). The uncleaved signals can span the membrane in different orientations, dictated by the positive inside rule (von Heijne, 1986b; von Heijne, 1999). If there are positive charges preceding the hydrophobic stretch, then the transmembrane (TM) segment is oriented with the C-terminus facing the periplasm whereas if the hydrophobic stretches are followed by positively charged residues, then the amino-terminus of the TM segment is localized to the cytoplasm. The positive inside rule is based on the finding that the membrane proteins have cytoplasmic loops that are enriched in positively charged residues (Lys, Arg) as compared to the periplasmic/translocated loops (von Heijne, 1986b).

### 2.1 Signal Peptide Targeting to the Membrane

**FIGURE 2:**
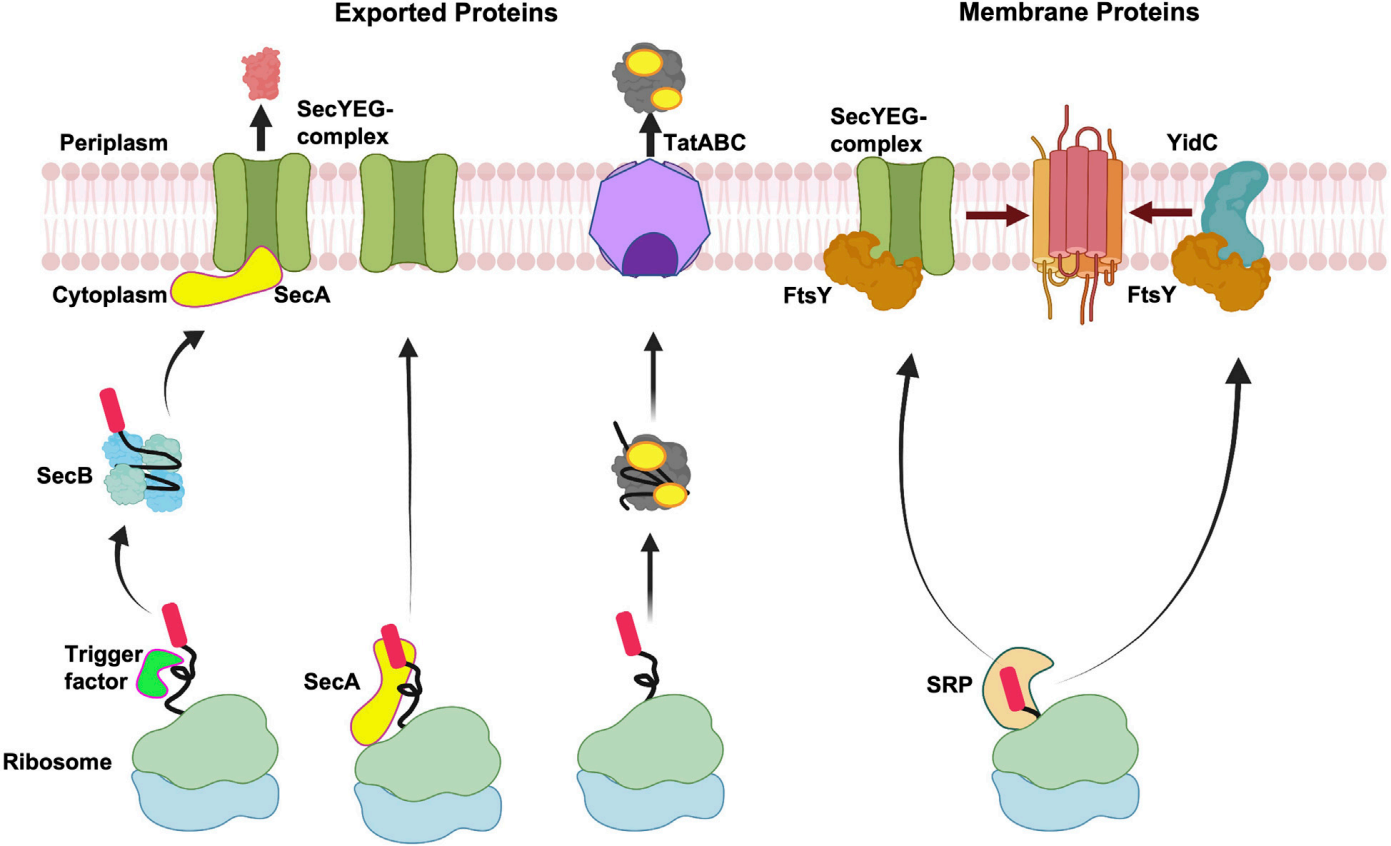
Membrane targeting pathways. Overview of targeting of exported proteins and membrane proteins. After exported proteins are released from the ribosome, Sec-dependent proteins can be stabilized by the molecular chaperone SecB in an unfolded state and then targeted to SecA at the membrane, followed by translocation by the SecYEG complex. Alternatively, SecA can interact with the ribosome bound nascent chain and target the exported protein to the SecYEG complex. In case of Tat complex, the proteins fold in the cytoplasm before being exported by the Tat complex. In the event of co-translational targeting, the nascent membrane proteins form a complex with SRP, which target proteins to FtsY (SRP receptor) for membrane insertion either by the SecYEG complex or the YidC insertase. Created with BioRender.com.

Targeting of exported and membrane proteins is initiated early on after the amino terminus of the nascent protein emerges from the ribosomal exit channel (Figure 2). The targeting pathway is decided by the interaction of the nascent protein with the ribosome-bound chaperones and targeting factors such as the Trigger Factor (TF) (Hoffmann et al., 2010; Castanié-Cornet et al., 2014), the signal recognition particle (SRP) (Grudnik et al., 2009; Akopian et al., 2013; Saraogi and Shan, 2014) and SecA (the ATPase motor of the Sec translocation machinery) in some cases (Kusters and Driessen, 2011; Chatzi et al., 2014). These chaperones and targeting factors facilitate the localization to the inner membrane of bacteria.

In Gram-negative bacteria, the exported proteins are typically targeted to the Sec complex or the Tat translocase by the post-translational mechanism (Figure 2). Exported proteins which employ the Sec pathway contain moderately hydrophobic signal sequences and are transported through the Sec channel in a largely unfolded state. In contrast, the Tat substrates are translocated in the folded state after release of the protein from the ribosome and hence post-translational. Typically, the integral membrane proteins are targeted co-translationally either to the Sec machinery or the YidC insertase as soon as the hydrophobic TM segment emerges from the ribosomal tunnel.

#### 2.1.1 Targeting of Exported Proteins

##### 2.1.1.1 Sec Proteins

In the post-translational pathway, the TF is bound to the ribosome over the exit channel shielding the nascent chains from proteases (Figure 2) (Ferbitz et al., 2004). The ribosome-bound TF provides a protective environment preventing the premature folding and aggregation of the growing protein chain. Ribosome profiling studies have shown that the TF binds to the nascent chain only after approximately 100 amino acids are synthesized and play a role for the biogenesis of many β-barrel outer membrane proteins (Oh et al., 2011). After the protein is released from the ribosome, some proteins can form a complex with SecB (Kumamoto and Francetić, 1993), a dedicated molecular chaperone for export in bacteria. SecB is a tetramer (Xu et al., 2000) and how it keeps proteins in a non-native loosely-folded form (Randall and Hardy, 1986) is an intriguing mechanistic question. Recently, a state-of-the-art NMR study revealed that an unfolded preprotein wraps around the SecB. This is achieved by binding to the long hydrophobic grooves of SecB that run around the tetramer (Huang et al., 2016). The SecB delivers the preprotein to SecA bound to SecYEG at the membrane (Hartl et al., 1990). The targeting of the preprotein to SecA is achieved by SecA acting as a receptor that binds the signal peptide (Gelis et al., 2007) and the chaperone SecB (Zhou and Xu, 2003).

In an alternative scenario, cytosolic SecA can interact with the nascent chains emerging from the ribosome (Figure 2). Indeed, previous studies had suggested that SecA interacts with the nascent chains (Chun and Randall, 1994; Karamyshev and Johnson, 2005). In more recent studies, SecA was shown to interact with the ribosome near the ribosome exit channel (Huber et al., 2011). The binding of SecA to the ribosome is mediated by the ribosomal protein, L23. This interaction is important since mutations in L23 perturb SecA ribosome binding, significantly affecting the post-translational export of proteins *in vivo* (Huber et al., 2011). The isolation of mRNAs that copurify with SecA revealed that they encode both Sec exported and membrane proteins (Huber et al., 2017). The interaction of SecA with the nascent protein chains occurs only when the chains are longer than 110 residues. The interaction of SecA with the nascent chains is not dependent on TF or SecB. Notably, the authors found that SecB interaction with the nascent chains depended on SecA being bound to the nascent chains, suggesting that SecA interacts with a subset of SecB dependent substrates co-translationally (Huber et al., 2017). The emerging data suggests that SecA bound nascent chains can target proteins directly to the SecYEG complex or with the help of SecB (Figure 2). However, it is uncertain if the interaction of SecA with all SecA-dependent substrates occurs co-translationally.

In some cases, the preprotein is released from the ribosome with the TF still bound (not shown in Figure 2). *In vitro,* TF has been shown to form a stable 1 to 1 complex with proOmpA (Crooke et al., 1988), making proOmpA translocation competent. The TF functions as a holdase and foldase to bind its substrate in an unfolded state (Hoffmann et al., 2010). Saio et al. (2014) characterized the binding of the TF to the unfolded precursor of alkaline phosphatase (pre-PhoA) by NMR. With the help of multiple binding pockets, the TF engages with the nascent polypeptide and shields the emerging hydrophobic regions of pre-PhoA in solvent to prevent it from premature folding and aggregation. De Geyter et al. (2020) showed that the TF is a genuine export chaperone. Notably, they revealed that the TF bound preprotein can associate with the SecB, which then recruits SecA through its C-tail and promotes the transfer of the preprotein to SecA.

##### 2.1.1.2 Tat Proteins

A different post-translational mechanism is used for targeting of Tat proteins to the inner membrane (Figure 2). These Tat proteins need to be folded in the cytoplasm prior to their translocation across the membrane (Palmer and Stansfeld, 2020). Many of the known substrates of the Tat pathway in bacteria bind a variety of redox cofactors, including molybdopterin centers and FeS cluster. There are specialized chaperones termed REMP (redox enzyme maturation proteins) to mediate cofactor insertion and proof reading (Turner et al., 2004; Robinson et al., 2011). For example, TorD is a REMP for TorA that encodes Trimethylamine-N-Oxide Reductase. TorD facilitates cofactor insertion and protects the TorA signal peptide from proteases (Ilbert et al., 2003) enabling the TorA to be delivered correctly to the Tat translocase (Jack et al., 2004). Another REMP is the DmsD that is involved in the biogenesis of dimethyl sulphoxide (DMSO) reductase (DmsA) (Ray et al., 2003). DmsD associates with the DmsA signal peptide (Oresnik et al., 2001) and also interacts with the molecular chaperones DnaK, DnaJ, GroE, GroEL, and TF (Li et al., 20101804; Castanié-Cornet et al., 2014). Finally, NapD is a REMP for the nitrate reductase complex localized in the periplasmic space. NapD binds to the Tat signal peptide of NapA (Maillard et al., 2007) and is involved in the insertion of the molybdenum cofactor.

#### 2.1.2 Targeting of Membrane Proteins

For integral membrane proteins the hydrophobic segments in the nascent proteins interact with SRP at the ribosome exit channel and are sorted away from exported proteins that contain less hydrophobic sequences (Figure 2) (Lee and Bernstein, 2001). The inference for this comes from ribosome profiling studies examining the mRNAs that are bound to SRP engaged ribosome nascent chains (Schibich et al., 2016). The study revealed 87% of the SRP interactors are membrane proteins and only 6% are periplasmic/outer membrane proteins (Schibich et al., 2016). SRP can scan the ribosome with low affinity even before the nascent chain reaches the exit tunnel and interacts with the ribosomal binding proteins L23 and L29. This is called the stand-by mode (Holtkamp et al., 2012). When the nascent chain of 30–35 amino acids length reaches the exit site, SRP forms a high affinity complex with the translating ribosome and signal peptide (Bornemann et al., 2008; Holtkamp et al., 2012). Soon after forming this high affinity complex, the nascent chain is delivered to its receptor at the membrane (Figure 2, right side) to form a quaternary complex. The receptor FtsY (in prokaryotes) then transfers the ribosome nascent chain to the SecYEG complex by a mechanism involving the catalysis of GTPases.

The SRP has also been shown to target membrane proteins to the YidC insertase (Welte et al., 2012) (Figure 2). For example, MscL (Facey et al., 2007) and the tail anchored proteins SciP, DjlC, and Flk require both YidC and SRP for membrane protein insertion (Pross et al., 2016; Peschke et al., 2018). Ffh and FtsY can be crosslinked to the cytoplasmic loop of YidC, suggesting that the SRP-YidC nascent chains are targeted to FtsY that is in proximity to the YidC cytoplasmic loop (Petriman et al., 2018). The YidC cytoplasmic loop C2 and the C-tail of YidC binds to the ribosome supporting YidC activity (Geng et al., 2015).

Although the classical model predicts that SRP binds to the TM segment when it is exposed out of the ribosome exit channel, SRP can also interact with the hydrophobic regions that are not a part of the TM segment in some cases. Pross and Kuhn (2020) proved that there are two hydrophobic segments in the amino-terminal part of the C-tailed anchored protein SciP, which are recognized by SRP allowing it to target SciP to YidC . Additionally, in contrast to the classical view, ribosome profiling studies showed that 29% of the SRP interactors skipped interaction with the first TM segment of the membrane protein but were bound to C-terminal TM segments (Schibich et al., 2016). The SRP prefers to bind to ribosomes exposing  ~ 12-17 amino acids enriched in hydrophobic and/or aromatic residues (Ile, Leu, Val, Met, Phe, Tyr, Typ) (Schibich et al., 2016).

In another variation, certain membrane proteins with internal TM segment can be co-translationally targeted to the membrane by SecA (Wang et al., 2017). SecA binds to the ribosome near the exit channel where it can recognize certain membrane proteins. SecA interacts with high specificity with nascent RodZ chains containing a TM segment far from the amino-terminus and targets the protein to the inner membrane (Wang et al., 2017). Previously, Rawat et al. (2015) had shown that SecA drives TM insertion and that Ffh and FtsY were not involved. SecA is sufficient for membrane targeting of RodZ both *in vivo* and *in vitro* (Wang et al., 2017). Interestingly, Knüpffer et al. (2019) found that SecA, just like SRP, deeply inserts into the exit tunnel of the ribosome to make contact with the intra-tunnel loop of L23 (Knüpffer et al., 2019). When the nascent chain is synthesized, SecA withdraws from the tunnel and the SecA bound to the L23 ribosome protein recruits the nascent TM segment. It is intriguing that the SecA amino-terminal amphipathic helix and the ribosomal L23 protein bind the nascent chain TM segment with the TM segment clustered in between, as revealed by Cro-EM studies (Wang et al., 2019). The SecA ribosome nascent chain complex is then targeted to the SecYEG complex, which repositions SecA on the ribosome, allowing the TM segment containing the nascent chain to be handed over to the SecYEG.

### 2.2 Crossing the Membrane

**FIGURE 3:**
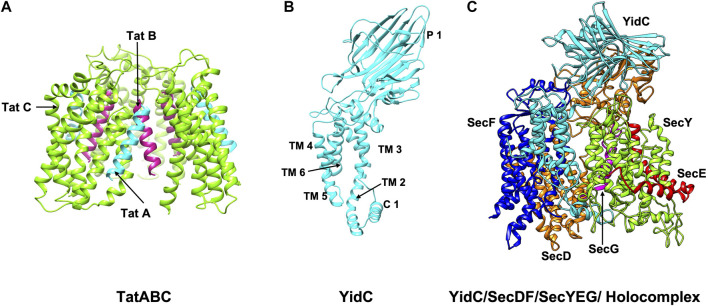
The structures/models of the bacterial export and insertion machineries. Export of proteins across the membrane are catalyzed by **(A)** the Tat complex (resting complex shown) in a folded state (left side) (Habersetzer et al., 2017) or **(C)** by SecYEG/SecDF/YidC [adapted from Botte et al. (2016) PDB: 5MG3] energized by the SecA motor ATPase (not shown) in an unfolded state. TatA, TatB and TatC is shown in cyan, magenta and green, respectively. SecY, SecE, and SecG is shown in green, red, and magenta; SecD, SecF and YidC are shown in orange, blue and cyan. Membrane protein integration is catalyzed by the SecYEG/SecDF/YidC **(C)** complex or by the YidC insertase **(B)** [adapted from Kumazaki et al. (2014b) PDB: 3WVF]. The view is in the plane of the membrane with the periplasmic face at the top and the cytoplasmic face at the bottom.

Once the signal peptide has navigated the transported protein to the membrane, it promotes interaction with the translocation machineries (see below). A vast majority of proteins are translocated by the SecYEG/SecDF system (Figure 3C) and SecA (Oliver and Beckwith, 1981; Crane and Randall, 2017; Tsirigotaki et al., 2017; Cranford-Smith and Huber, 2018), whereas the Tat machinery is involved in the export of around 30 proteins in *E. coli* (Berks, 2015; Palmer and Stansfeld, 2020) (Figure 3A). As mentioned before, the Tat machinery is radically distinct from the SecA/SecYEG/SecDF system as it can export fully folded proteins.

The SecYEG/SecDF/YidC translocase (Figure 3C) plays the principal role for placing membrane proteins in the lipid bilayer with the correct topology (Cymer et al., 2015). Additionally, it functions to translocate hydrophilic domains of membrane proteins across the membrane and allows the hydrophobic regions to integrate into the lipid bilayer. The YidC insertase on its own or in cooperation with the Sec translocase can insert membrane proteins (Kiefer and Kuhn, 2018) (Figures 3B,C). The Tat machinery can act as insertase for the membrane proteins with C-terminal TM segments (Palmer and Stansfeld, 2020).

#### 2.2.1 Protein Translocation Across the Membrane

##### 2.2.1.1 SecYEG/SecA Translocase

SecA plays a crucial role for the export process both as a receptor and molecular motor (Cranford-Smith and Huber, 2018). The preprotein binds with high affinity to SecA/SecYEG but not to SecYEG (Hartl et al., 1990). SecA is also necessary for the translocation of proteins across the inner membrane (Oliver and Beckwith, 1981).

**FIGURE 4:**
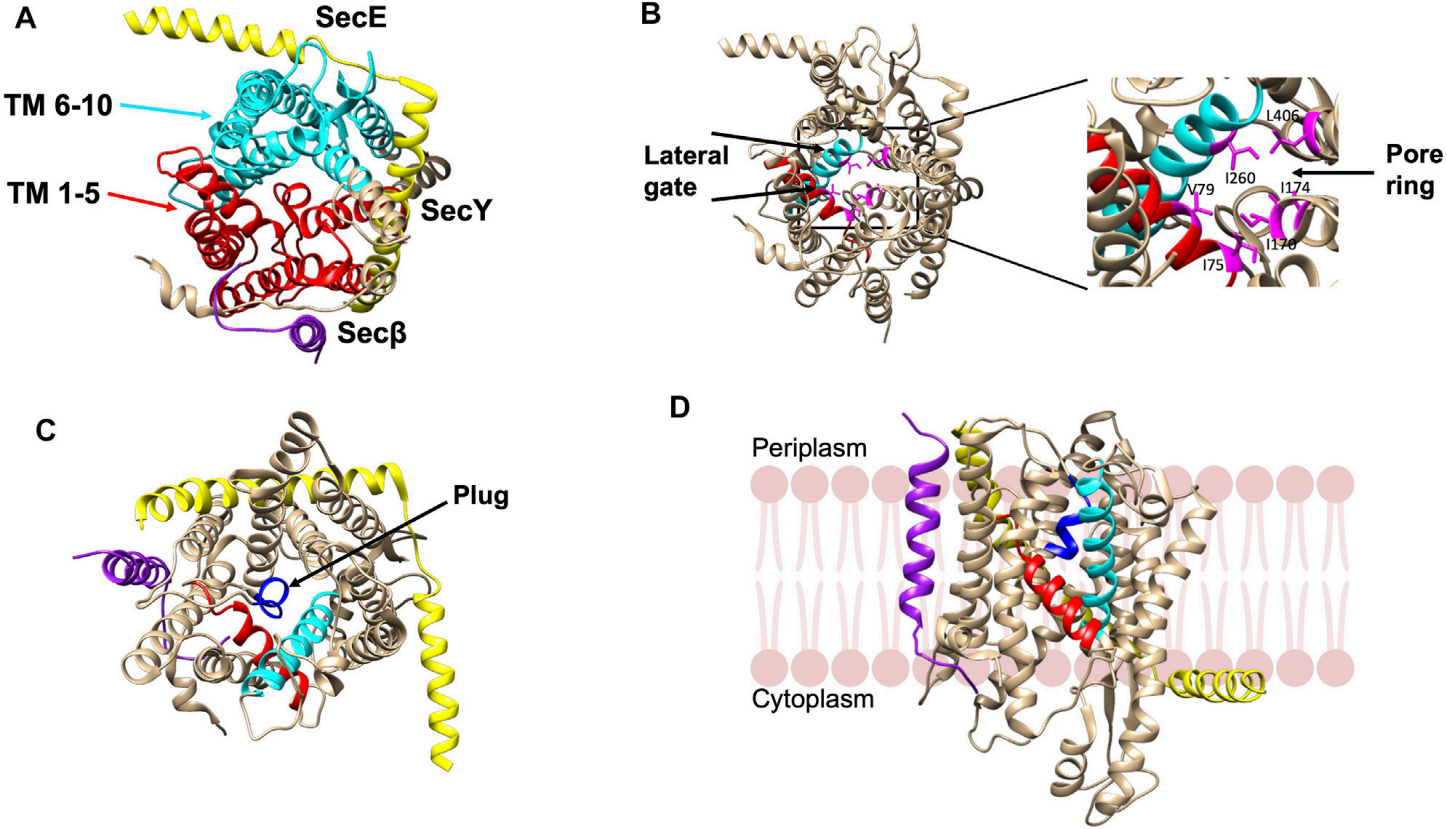
Crystal structure of the SecYEβ complex in the resting state from *Methanocaldococcus jannaschii* [adapted from van den Berg et al. (2004) PDB: 1RHZ] **(A)**. TM1-5 (red) and TM6-10 (cyan) are the halves of SecY. SecE and Sec61β are in yellow and purple, respectively. **(B)** The pore ring comprised of six residues (pink) and lateral gate (TM2b in red and TM7 in cyan) are highlighted. **(C)** The plug helix located above the pore ring is indicated in dark blue. **(D)** The SecYEβ complex from *Thermus thermophiles* (PDB: 5AWW). The lateral gate region comprised of TM2b (red) and TM7 (blue) is the site where the signal peptide or TM segments of membrane proteins exit the channel upon opening of the gate. The SecYEβ structures in **(A–C)** are shown perpendicular to the membrane.

The structure of the SecYEβ protein-conducting channel, comprised of three subunits, was solved from *Methanococcus jannashii* in 2004 (van den Berg et al., 2004). SecY is the main channel forming unit that has a classic hourglass structure where TM 1–5 and TM 6–10 form two symmetric bundles held together by a linker (Figure 4A). The second subunit, SecE forms a clamp around SecY by wrapping around the two sides *via* its TM segment and cytoplasmic tail to stabilize the complex (Figure 4A). The SecE in *E. coli* is a 14 kDa essential 3TM protein. Sec61β (SecG of *E. coli*) is located on the third side of SecY (Figure 4A). Both SecY and SecE are evolutionarily conserved in bacteria, archaea and eukaryotes while SecG is not conserved in the three domains of life (Hartmann et al., 1994; Pohlschröder et al., 1997).

There is a pore ring with a diameter of 4—6 Å (Figure 4B) at the center of the SecY channel (van den Berg et al., 2004). The pore ring is formed by 6 hydrophobic aliphatic residues and expands to accommodate the polypeptide chain during translocation (Bonardi et al., 2011). A short helix TM 2a termed as the “plug” keeps the pore closed (Figure 4C). The plug functions to maintain the integrity and preserve the permeability barrier of the membrane (Li et al., 2007). It has been shown that deleting the plug domain does not result in a major defect in protein export. However, channel experiments have shown that deletions of the plug compromise the membrane permeability of the channel as there are fluctuations between the open and closed state of the translocon (Li et al., 2007). This suggests that when the plug is present, the channel is stabilized in the closed state. Finally, on the front side of the channel, is the lateral gate (comprised of the TM 2a and TM 7) (Figure 4D) that can open sideways to allow signal peptides or TM segments to exit the channel (van den Berg et al., 2004).

**FIGURE 5:**
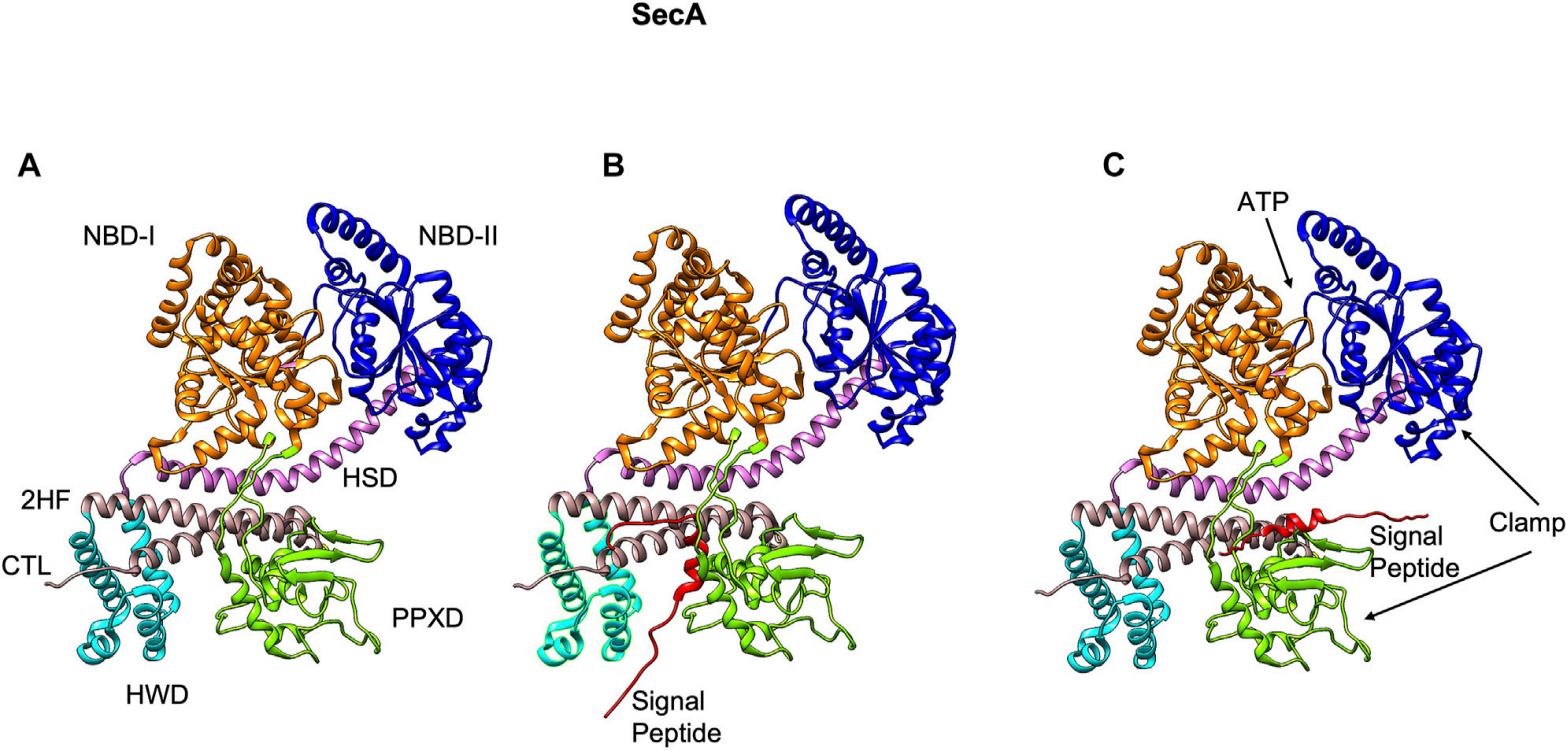
The NMR structure of SecA from *E. coli* [adapted from Gelis et al. (2007) PDB: 2VDA]. **(A)** The various domains of SecA are highlighted (without the signal peptide). The nucleotide binding domains I (orange) and II (blue), the central helix subdomain of helical scaffold domain (HSD in purple), the preprotein crosslinking domain (PPXD green), the helical wing domain (HWD cyan), and the observed carboxyl-terminal linker domain (CTL). Also highlighted is the 2-helix finger (2HF tan) within the HSD domain. **(B)** The signal peptide (red) binds roughly perpendicular to 2HF based on NMR studies (Gelis et al., 2007). **(C)** The signal peptide is modeled parallel to the 2HF of the *E. coli* SecA NMR structure based on FRET, mutagenesis and genetic studies (Grady et al., 2012).

The peripheral subunit of the Sec complex is SecA which docks onto the SecYEG channel. It utilizes the energy from both ATP binding as well as ATP hydrolysis to drive the transport of unfolded proteins across the Sec channel. Structurally, SecA contains multiple domains with two ATP binding domains (NBD-1 and NBD-2) (Hunt et al., 2002), the HSD (helical scaffold domain), a preprotein crosslinking domain (PPXD) (Hunt et al., 2002), a helical wing domain (HWD), and a carboxyl-terminal linker domain (CTL) (Figure 5A). The HSD domain also has the central helix and the 2 helix finger (2HF) (Zimmer et al., 2006) or the regulator of ATPase (IRA1) (Karamanou et al., 1999) subdomains. PPXD and the 2HF have been implicated in binding the signal peptide and the mature region of the preprotein (Kourtz and Oliver, 2000; Papanikou et al., 2005; Musial-Siwek et al., 2007).

**FIGURE 6:**
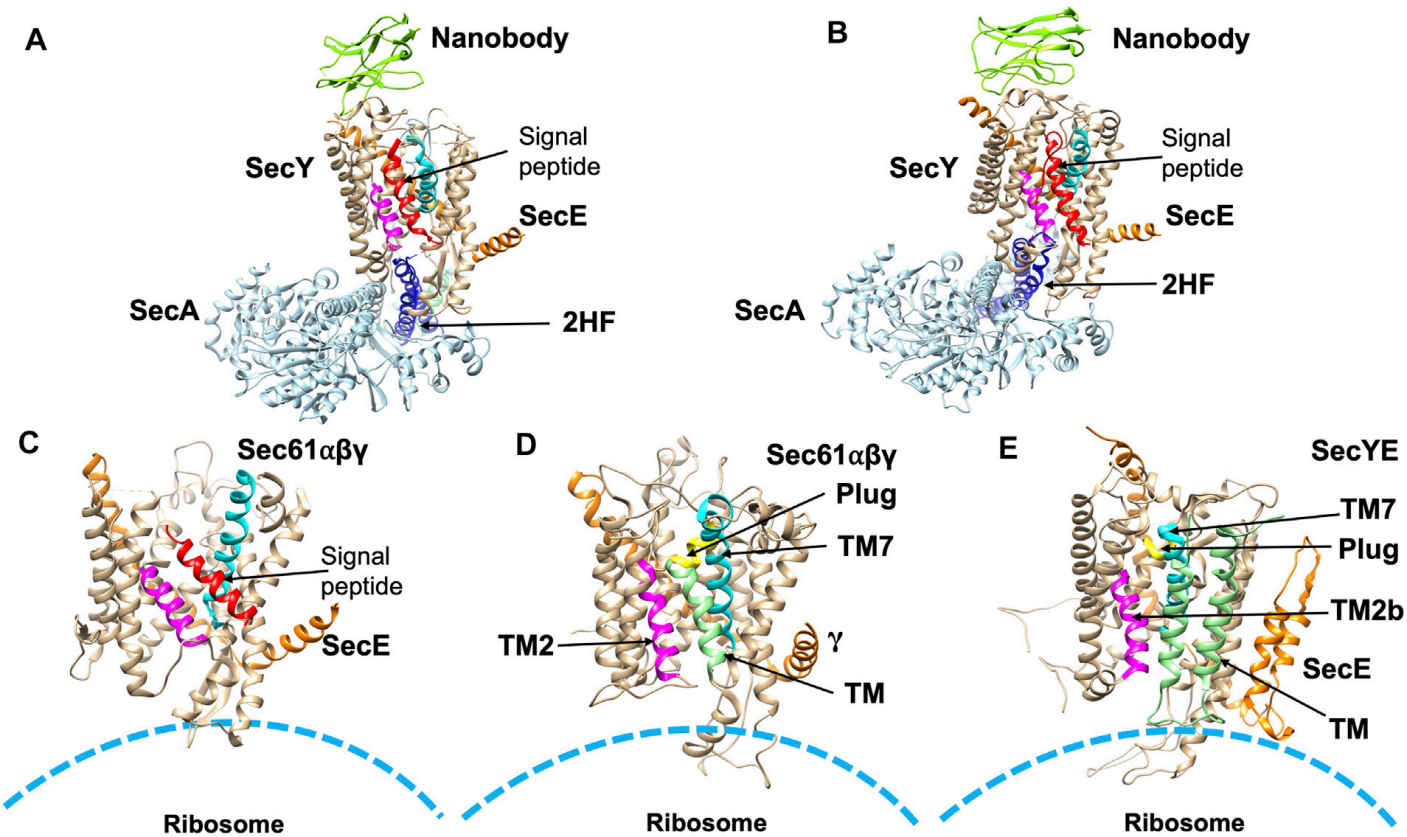
Structures of substrate engaged SecYE or Sec61 complexes. **(A)** Crystal structure of SecYE-SecA [adapted from Li et al. (2016) PDB: 5EUL] with a portion of the preprotein (comprised of the OmpA signal sequence and a few residues in the mature region) fused into the 2HF (navy blue) by insertion between 741 and 744 of SecA. SecA (in light blue) was from *B. subtilis* and SecYE was from *Geobacillus thermodenitrificans*. Nanobody (chartreuse) bound to the periplasmic side of SecY (tan). **(B)** CryoEM structure of SecYEG-SecA complexed with a proOmpA sfGFP [adapted from Ma et al. (2019) PDB: 6ITC] fusion protein. The structure was performed with SecYE in a lipid nanodisc. An anti-GFP nanobody was inserted at the C-terminus of SecA to recognize and stabilize the fused sfGFP of the substrate. In addition, a disulfide was created between a cysteine at position 8 in the early mature region of the proOmpA GFP fusion protein and a cysteine placed in the plug domain of SecY. Finally, a SecY nanobody that recognizes the periplasmic SecY region was added to stabilize the complex. SecA was from *B. subtilis* and SecYE was from *Geobacillus thermodenitrificans*. The nanobody is shown in green in **(A,B)**. **(C)** CryoEM structure [adapted from Voorhees and Hegde, 2016 PDB: 3JC2] in detergent of the canine ribosome Sec61 channel engaged with the N-terminal 86-amino acid preprolactin region. **(D)** CryoEM structure [adapted from Gogala et al. (2014) PDB: 4CG6] of the canine Sec61 channel engaged with a hydrophobic TM segment (light green) of a leader peptidase (lep) arrested intermediate. The TM segment was modeled within the opened TM2/TM7 lateral gate. **(E)** CryoEM structure (adapted from Bischoff et al. (2014) PDB: 5ABB) of a stalled *E. coli* ribosome SecYE complex engaged with proteorhodopsin (TM indicated in light green). TM2 and TM7 of the lateral gate are shown in magneta and cyan, respectively in **(A–E)**. The signal peptide (red) is indicated in **(A–C)**. The plug helix is indicated in yellow in **(D,E)**.

The crystal structures of the SecA-SecYEG complexes with and without substrate have shed light on how SecA moves the substrate polypeptide through the channel (Zimmer et al., 2008; Li et al., 2016; Ma et al., 2019). In the crystal structure of the SecA–SecYEG complex, a single SecA protein is bound to a single SecYEG protomer creating a groove for the passage of the preprotein (Zimmer et al., 2008). A clamp region can be observed at the interface of PPXD and NBD-2 domains (Figure 5A). Based on the crosslinking studies, the clamp has been proposed to bind the preprotein (Bauer and Rapoport, 2009). The 2HF region of SecA (Figure 6A) may push the preprotein into the channel (Bauer and Rapoport, 2009). Interestingly, while an NMR study (Gelis et al., 2007) showed the signal peptide was bound to a SecA groove formed at the interface of the 2HF and the PPXD (Figure 5B), it is possible that it would move from this region to align more parallel to the 2HF, such that it could be pushed into the channel. Indeed, based on the FRET, mutagenesis and genetic studies, Oliver and coworkers proposed a model where the signal peptide binds parallel to the 2HF (Figure 5C) (Grady et al., 2012).

To examine the structure of the SecY channel during active SecA-dependent translocation, X-ray crystallography was used to solve a substrate engaged SecA-SecYE complex (Figure 6A). The substrate sequence, which included the OmpA signal sequence and a short mature region, was inserted at the end of the 2HF of SecA (Li et al., 2016). The structure suggested that the signal peptide moved into the lateral gate facing the lipid bilayer and the mature region inserted into the channel as a loop, displacing the plug. Thus, the interaction of the signal sequence with the lateral gate induces conformational changes and movements of the plug domain. This leads the way for the substrate to move up the pore ring towards the periplasm by repeated ATP binding and hydrolysis events moving roughly 20–25 amino acids into the translocon in each step.

More recently, in order to gain insight into the path of a translocating polypeptide through SecA, another substrate engaged SecA-SecYE was solved in an active transition state of ATP hydrolysis with ADPBeFx bound (Figure 6B) (Ma et al., 2019). The SecA/SecYE translocation intermediate with SecA locked in an ATP bound state was generated using a substrate fusion protein consisting of the proOmpA signal sequence, a linker region, and a folded GFP. In order to stabilize the complex, a cysteine was added after the signal sequence to form a disufide bond to the cysteine introduced at the SecY plug. The protein was then reconstituted into nanodiscs and solved by cryo-EM with a resolution of about 3.5 Å. Tracing the substrate within the SecA-SecYE complex confirmed that in addition to the polypeptide being in proximity to the SecA 2HF, it also interacts with the SecA clamp region via a short β-strand. It also showed that the signal sequence forms a helix that is positioned in a groove outside the lateral gate of the SecY channel.

There are several models that have been proposed to account for the role of ATP energy in energizing SecA/SecYEG in protein transport, including (Hegde and Bernstein, 2006) power stroke, (Oswald et al., 2021), Brownian ratchet, (Frain et al., 2019), push and slide, and other mechanisms. According to the power stroke model, the SecA ATP hydrolysis causes conformational changes that result in mechanical pushing of the polypeptide chain through the SecYEG channel. Indeed, a large segment of SecA was proposed to move through the SecYEG channel to the periplasmic region in order to translocate the polypeptide to the trans side of the membrane (Ulbrandt et al., 1992; Economou and Wickner, 1994). Later versions of the power stroke model proposed that the 2HF, which is positioned at the entrance of the SecYEG channel, functions as a piston to push the polypeptide through the membrane. This rationale comes from the fact that the SecA 2HF interacts with the preprotein during protein translocation (Erlandson et al., 2008). Upon ATP binding, the 2HF undergoes a large conformational change that pushes the protein substrate chain into the SecY channel (Catipovic et al., 2019). After the 2HF releases the polypeptide substrate of the preprotein, the finger resets to its original position (Catipovic et al., 2019). This cycle of conformational changes occur multiple times until the polypeptide is translocated through the channel.

In a Brownian ratchet mechanism, the movement of a protein chain occurs *via* diffusion through the channel. SecA would mediate SecYEG channel opening thereby enabling the preprotein to diffuse through the SecYEG pore. The evidence for this action was presented in a model by Allen et al. (2016). The authors demonstrated that the SecYEG gate is wide open when ATP is bound to SecA and slightly open with ADP bound to SecA. The slightly open channel allows protein substrate regions with small side chains to slide through the pore, but larger side chains would require the pore to open. Interestingly, the SecYEG channel and the SecA 2HF are able to detect the presence of a protein chain which results in nucleotide exchange, allowing ATP to replace ADP. The binding of ATP to SecA leads to opening of the SecYEG channel through which the chain crosses by diffusion. Backsliding of the polypeptide chain is prevented by closure of the channel. More recently, ATP-driven translocation through the SecYEG channel was shown to be indirectly coupled to ATP hydrolysis providing further support to the Brownian ratchet model (Allen et al., 2020).

A push and slide mechanism combines the power strokes and the passive diffusion models. Bauer et al. (2014) found that certain protein chains can slide passively through the SecYEG channel without ATP hydrolysis. Passive sliding of the polypeptide chain takes place after the preprotein is released by the 2HF and SecA has bound to ADP. Under these conditions, the clamp region between PPXD domain and NBD-2 domain is open and cannot bind the mature domain. The polypeptide chain can passively slide in either direction. Power stroke would occur again after the binding of ATP to SecA. During the power stroke, segments of the polypeptide chain move deep into the SecY channel (Catipovic and Rapoport, 2020) as the SecA 2HF moves into the channel. Prior to the retraction of the 2HF to the original position, the SecA captures and tightens its clamp region around the mature domain of the preprotein substrate, thus preventing back sliding of the polypeptide chain (Catipovic et al., 2019). This tightening enables the forward translocation of the chain to be maintained. The clamp closure occurs before or during SecA ATP hydrolysis and that the 2HF resets all the way when the clamp is closed. Otherwise, as the 2HF resets, and moves away from the channel it would drag the polypeptide with it (Catipovic et al., 2019; Catipovic and Rapoport, 2020). One baffling fact is that the immobilization of the 2HF to SecYEG do not inhibit translocation (Whitehouse et al., 2012).

In addition to the energy of ATP binding and hydrolysis, the proton motive force (pmf) can also contribute to the translocation of preproteins across the SecYEG channel membrane (Date et al., 1980; Zimmermann and Wickner, 1983). SecD and SecF, which have 6 TM segments and a large periplasmic domain, are required for pmf stimulation of protein translocation (Arkowitz and Wickner, 1994). One model proposes that SecDF assists in translocation by binding the preprotein once it emerges from SecY and prevents back sliding (Tsukazaki et al., 2011). Then it swivels its head domain to translocate about 25 amino acids across the membrane with the help of the pmf. Therefore, SecDF functions in the pulling of preproteins across the membrane, and the release of preproteins from the SecYEG complex after complete translocation.

##### 2.2.1.2 Tat Translocase

The Tat machinery exports fully folded proteins of different sizes. It is comprised of TatA, TatB, and TatC (Tat complex) in *E. coli* and TatAC in *B. subtilis* (Jongbloed et al., 2004; Berks, 2015; Palmer and Stansfeld, 2020). The Tat components in *E. coli* are assembled on the cytoplasmic membrane as a TatBC complex and a cytoplasmic TatA pool. Interestingly, TatC membrane insertion is a SecYEG and YidC dependent event (Welte et al., 2012; Zhu et al., 2012). After forming the TatBC complex, the TatA oligomers are recruited to the TatBC complex in a pmf dependent event before substrate translocation. The recognition of the twin arginine motif by a conserved patch on TatC (Palmer and Stansfeld, 2020) initiates the architectural reorganization of the active complex assembly. The low stability, size and transient nature of the active complex presents a daunting challenge to identify the precise assembled active complex needed for Tat translocation.

The Tat components TatA and TatB have similar features each possessing a small periplasmic N-terminal region, a single short TM helix (TMH) followed by an amphipathic helix (APH), and a cytoplasmic domain. TatA is assumed to form the translocation complex with the substrate as it is capable of forming oligomeric rings of different sizes (Gohlke et al., 2005). TatB functions with TatC as a receptor to bind Tat substrates (Cline and Mori, 2001; Behrendt and Brüser, 2014). TatB and TatC form a 1:1 complex and have an oligomeric structure with a size range of 360–700 kDa (Bolhuis et al., 2001).

In the resting state, the TatBC receptor complex most likely has some TatA associated and recent studies suggest there are three to four copies each of TatA, TatB and TatC in a ratio of 1:1:1 (Bolhuis et al., 2001; Alcock et al., 2016; Habersetzer et al., 2017). Based primarily on crosslinking studies it has been proposed recently that in the resting state, this TatABC complex is organized such that TatB binds to the TatC TM5 and TatA binds to the TatC TM6. Upon activation of the complex by substrate addition, TatA and TatB switch positions which may be triggered by the substrate with the signal peptide moving further into the membrane interior (Habersetzer et al., 2017).

**FIGURE 7:**
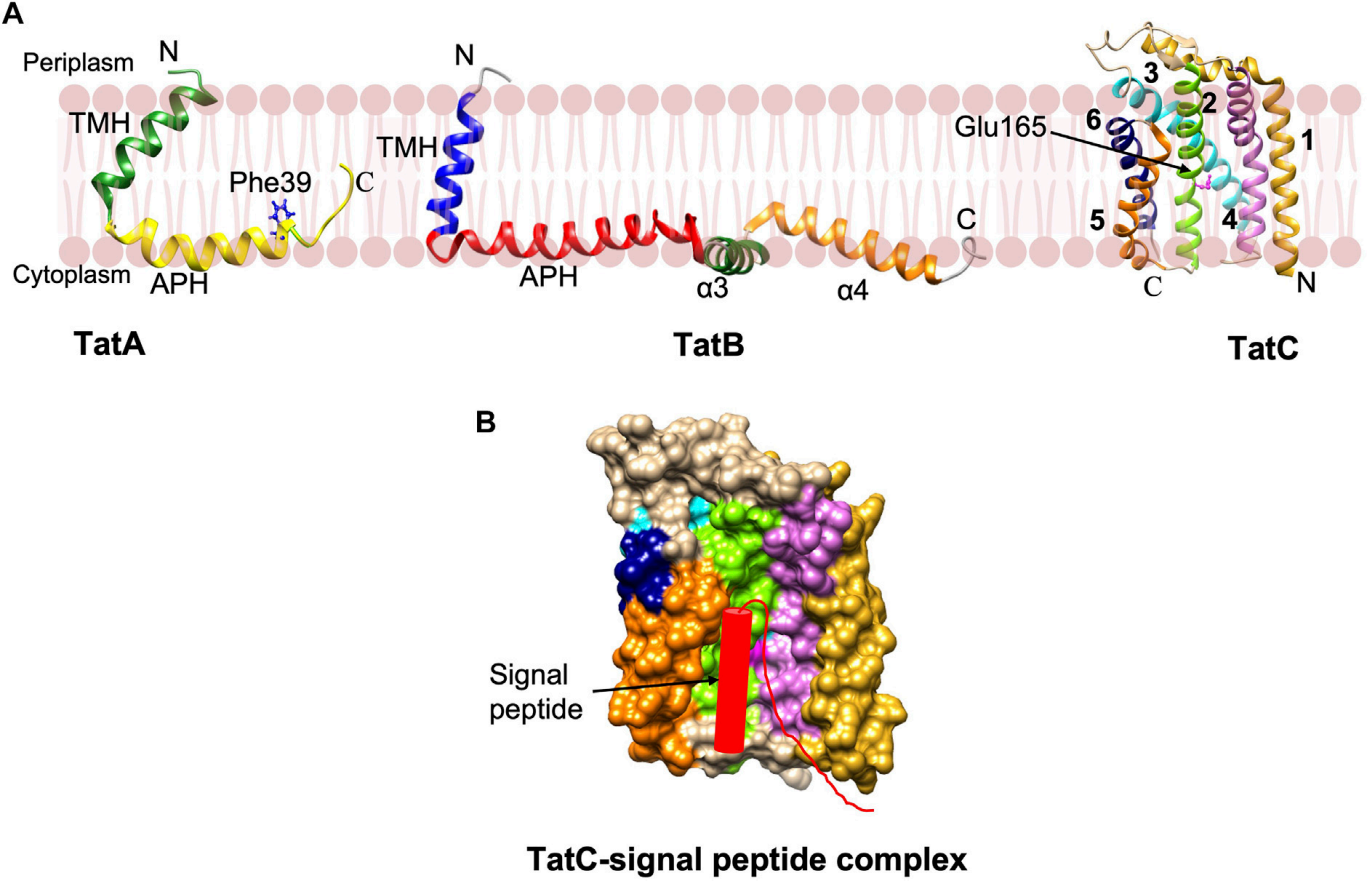
The Tat complex components and a model of TatC-signal peptide complex. **(A)** The single span TatA (PDB: 2MN7) and TatB (PDB: 2MI2) proteins were determined by NMR (Rodriguez et al., 2013; Zhang et al., 2014). The structure of 6 membrane spanning TatC [adapted from Rollauer et al. (2012) PDB: 4B4A] was solved by X-ray crystallography (Rollauer et al., 2012; Ramasamy et al., 2013). **(B)** The model of TatC binding with the Tat signal peptide in the groove adapted from Ramasamy et al. (2013). Only signal peptide and early mature region of the preprotein are indicated.

NMR studies reveal that TatA and TatB proteins possess a short α-helical TM segment followed by the amphipathic helix (APH) (Figure 7A) (Rodriguez et al., 2013; Zhang et al., 2014). TatA forms oligomeric rings with variable number of TatA molecules (Rodriguez et al., 2013). Interestingly, structural studies and molecular dynamic (MD) simulations predict that the TatA structure causes significant thinning of the membrane due to its short TM segments (Rodriguez et al., 2013). This may be true with TatB as well since it has a short TM segment. The structure of the 6-membrane spanning TatC from *Aquifex aeolicus* revealed that the protein has a “cup hand” or “glove-like structure” (Figure 7B), where TatC assembles into a concave structure that can accommodate a TM segment of TatB or the neighboring TatC (Rollauer et al., 2012; Ramasamy et al., 2013). Remarkably, the TatC surface has an ionizable Glu165 side chain that is expected to be buried in the hydrophobic interior of the bilayer. MD simulations show that the TatC has a hydrated cavity facing the cytosol with the conserved Glu/Gln at this position inside the membrane. This hydrophilic cavity and the short TM segments 5 and 6, cause thinning of the membrane.

Export by the Tat pathway begins by the recognition of the Tat signal peptide of the preprotein substrate by TatC within the TatBC complex. TatC recognizes the RR motif via its N-terminal domain and a cytoplasmic loop 1 (Figure 7B) (Alami et al., 2003; Gérard and Cline, 2006). Subsequently, insertion of the signal sequence into the interior of the membrane takes place by contacting the periplasmic side of TatA. Following the substrate insertion into the TatBC complex, the oligomeric TatA complex is formed in a step that requires a TM pmf. An oligomeric complex of TatA facilitates the translocation of the folded substrate. TatA protomers are predicted to form oligomeric ring-like pores of varying diameters in the cytoplasmic membrane, permitting the movement of fully-folded proteins into the periplasm (Lausberg et al., 2012).

The precise mechanism of translocation is still debatable, but we will discuss the two main hypotheses documented in literature. The trap door model postulates that the amphipathic helix (APH) domain of TatA flips into the lipid bilayer with the help of membrane potential on contact with the substrate carrying the TatA interactive motif (Patel et al., 2014). Initially, TatA oligomers self-arrange to form a pore of ∼8.5–13 nm in diameter to accommodate the folded protein (Frain et al., 2019). The APH of the TatA oligomers at the cytoplasmic face mirrors a “trap door” that regulates the transient channel for translocation of the substrate. This would essentially depend on the flexible hinge (the conserved Gly residue) between the APH and TMH (Frain et al., 2019). When the APH swings up to align with the TMH, the polar residues may interact with the hydrophilic protein to be translocated and thus the folded protein is promoted across the formfitting passive conduit.

The second model proposed a weakening of the lipid bilayer when TatA oligomerizes with its polar N-tail destabilizing the membrane, allowing translocation of the Tat substrate (Brüser and Sanders, 2003). This model where transient bilayer disruptions occur, is gaining more support with the NMR structure of TatA and suggests that the TatA topology may not be as flexible as predicted by the trap door model. MD simulations reveal the phospholipids are highly distorted and the membrane thickness is dramatically shortened (Rodriguez et al., 2013). It is believed that the thinning of the membrane is due to the short TatA TM segment and the presence of the conserved glutamine in the oligomer. However, this model does not clarify what drives the translocation of the substrate across the membrane.

### 2.3 The Insertion of Proteins Into the Membrane

The insertion of proteins in their proper conformation and orientation into the lipid bilayer is crucial for the functional integrity of the membrane proteins [for recent reviews see (Cymer et al., 2015; Tsirigotaki et al., 2017; Hegde and Keenan, 2021)].

#### 2.3.1 SecYEG/YidC

For membrane protein topogenesis, the nascent membrane protein chain is presumed to be driven across the membrane utilizing the GTP hydrolysis energy from protein synthesis. This is possible because the ribosome binds to the SecYEG complex and may form a single aqueous conduit that stretches across most of the inner membrane.

As the membrane protein enters the SecYEG channel, the hydrophobic TM sequence or signal peptide may first enter the hydrophilic channel and then exit the lateral gate (van den Berg et al., 2004) or it can slide into the membrane via the lateral gate by thermodynamically partitioning between the lipid and the aqueous pore (Cymer et al., 2015). Rather than the sequence of amino acids of the TM segment, the overall hydrophobic character of the segment is important for insertion into the membrane (Hessa et al., 2005; Hessa et al., 2007). The hydrophobic stretch can stabilize the open lateral gate (Zhang and Miller, 2010).

As seen with the substrate engaged SecYEG/SecA complex, the ribosome bound-Sec translocon showed the signal peptide in the lateral gate region. A cryoEM structure of the canine ribosome Sec61 translocon engaged with a preprolactin substrate revealed the signal peptide in the lateral gate (Figure 6C) (Voorhees and Hegde, 2016). The pore region of the channel would allow the polar polypeptide chains to be translocated to the trans side of the membrane (Erlandson et al., 2008). After translocation, a simple membrane protein with 1 TM segment would completely exit the channel and partition into the lipid bilayer.

Similarly, the lateral gate accommodates the TM segment of the ribosome bound membrane protein inserting into the Sec61 complex, as revealed by cryo-EM study (Figure 6D) (Gogala et al., 2014). Notably, another cryo-EM study of a nascent membrane protein-SecYE complex demonstrated that the first 2 TM α-helices of proteorhodopsin had exited the lateral gate to face the lipid with the N-terminus at the periphery of SecY (Figure 6E) (Bischoff et al., 2014). Kater et al. (2019) further elucidated that a partially inserted hydrophobic region can cause opening of the lateral gate.

SecA is always required for translocation of large loops and occasionally for small loops of membrane proteins (Kuhn, 1988; Andersson and von Heijne, 1993; Deitermann et al., 2005; Soman et al., 2014). However, the mechanism by which this task is carried out has not been elucidated, as the ribosome is expected to be already bound to the SecYEG complex when the membrane protein inserts co-translationally. In order to perform the translocation function by SecA, the ribosome should be dissociated partly or completely from the SecYEG. This illustrates the dynamic nature of the insertion process and the interplay of the various SecYEG binding partners.

An interesting method to study *in vivo* insertion and co-translational folding of membrane proteins is the application of translational arrest peptides to measure forces acting on a nascent protein during membrane insertion (Ismail et al., 2012; Sandhu et al., 2021). In this approach, the arrest peptide binds to the ribosomal tunnel and induces ribosomal stalling at a specific amino acid. SecYEG mediated membrane insertion and folding of a nascent chain is followed by examining the release of stalling and resumption of protein synthesis. This technique has been used to study the co-translational insertion of simple to complex proteins spanning the membrane ten times, showing that the surface helices and re-entrant loops that flank a TM segment can either advance or delay membrane protein insertion (Nicolaus et al., 2021). Moreover, the results supported a sliding mechanism where the inserting TM segment moved into the membrane along the outer part of the lateral gate.

The mechanism of insertion of multispanning membrane proteins is complicated with most of the findings coming from studies of the endoplasmic reticulum (ER) membrane system (Cymer et al., 2015; Hegde and Keenan, 2021). In some cases, a TM segment is inserted into the translocase and then reoriented within the channel (Goder and Spiess, 2003). Remarkably, some TM segments of the membrane proteins such as the aquaporin 4 channel, exit the channel but apparently interact again at a later stage in membrane biogenesis, validating the dynamic nature of membrane protein biogenesis. The Sec machinery can handle the internal TM segments by various mechanisms. Some TM segments integrate into the lipid bilayer spontaneously (Heinrich et al., 2000), others integrate into the lipid bilayer only after the protein synthesis is terminated (Do et al., 1996; McCormick et al., 2003), some pairs of TM segments co-integrate into the membrane (Skach and Lingappa, 1993; Heinrich and Rapoport, 2003), while the rest can be stabilized by chaperones such as TRAM and YidC (Do et al., 1996; Heinrich et al., 2000; Beck et al., 2001; Urbanus et al., 2001; Nagamori et al., 2004).

SecYEG plays the primary role in membrane insertion in the plasma membrane in bacteria. The accessory component, YidC actively participates in membrane protein biogenesis for several different Sec dependent proteins (Figures 2, 3). Substrates that require the synergistic action of both YidC and SecYEG for insertion include, subunit a and b of the F1Fo ATP synthase (Yi et al., 2004) and TatC of the twin arginine translocase (Welte et al., 2012; Zhu et al., 2012). Moreover, YidC can bind to the TM segment of membrane proteins after the TM segment exits the SecYEG channel (Urbanus et al., 2001). This case is exemplified by the TM segments of FtsQ and leader peptidase (Houben et al., 2004) which were shown to initially contact SecYEG followed by contact with YidC at a later stage during its translocation process. This latter finding implied that YidC may facilitate Sec substrates to partition into the bilayer and assist in the clearing the Sec channel of its substrates. Remarkably, in the case of CyoA (subunit of cytochrome bo3 oxidase), the amino-terminal domain is inserted by the sole action of the YidC insertase whereas the large C-terminal domain requires SecYEG operating with the SecA motor ATPase for insertion (Celebi et al., 2006; van Bloois et al., 2006; Kol et al., 2009).

Furthermore, YidC acts as a chaperone (Nagamori et al., 2004) and assembler of multi-TM complexes (Saller et al., 2012). Studies with LacY biogenesis showed that YidC acts in the late stages of membrane protein biogenesis and is crucial for the correct folding of the protein but nonessential for its insertion (Nagamori et al., 2004; Zhu et al., 2013). Wagner et al. (2008) discovered a similar trend with MalF, a subunit of the maltose binding complex. Upon YidC depletion, the stability of the complex was affected without compromising the insertion of the TM segments of MalF mediated by the SecYEG complex.

In order to perform these multi-functions, YidC must be located close to the SecYEG complex. Indeed, YidC, SecDF/YajC may associate with SecYEG to form a holo-translocon (Schulze et al., 2014). This has been validated by copurification of YidC with the SecYEG and SecDF/YajC. The purified complex is capable of inserting *in vitro* synthesized membrane proteins (Komar et al., 2016). A low-resolution structure of the holocomplex SecYEG/SecDFYidC revealed that the SecYEG-YidC interface is a lipid filled cavity (Martin et al., 2019). Although a YidC holocomplex can be isolated, YidC is capable of dynamic interaction with SecYEG. When YidC contacts SecYEG, it is in proximity to the lateral gate of SecYEG and can contact helices on either side of the lateral gate (TMs 2b, 3, 7 and 8) (Sachelaru et al., 2017). This contact is maintained even in the absence of SecDF. Furthermore, SecYEG contacts TM1 and cytosolic loop 1 of YidC (Petriman et al., 2018). The Sec lateral gate can contact the YidC TM3-TM5 region which forms the greasy slide (Steudle et al., 2021). Taken together, these studies suggest that the insertion of Sec-dependent protein substrates occurs at the interface of YidC/SecYEG.

#### 2.3.2 YidC-Only Pathway

In addition to assisting SecYEG and acting as a chaperone for membrane insertion, YidC can also operate independently. Examples of the Sec-independent proteins include M13 phage coat protein (PC) and the Pf3 coat protein, which were earlier presumed to be inserted by an unassisted mechanism. Depletion of YidC resulted in the accumulation of these proteins in the cytoplasm (Samuelson et al., 2000; Chen et al., 2002; Serek et al., 2004). Moreover, crosslinking studies revealed that YidC interacts with the inserting Pf3 coat (Chen et al., 2002). Subunit c of F_1_F_0_ ATPase was shown to be dependent on YidC for membrane insertion both *in vivo* (Yi et al., 2003; van Bloois et al., 2004) and *in vitro* (Van Der Laan et al., 2004). Interestingly, YidC proteoliposomes were capable of forming the subunit c oligomer whereas the liposomes were not (Kol et al., 2006). The indispensable nature of YidC in cells is still speculative. One of the reasons may be attributed to the fact that YidC is required for the biogenesis of the respiratory complexes (van der Laan et al., 2003).

Other substrates for the YidC-only pathway are MscL (Mechanosensitive channel of large conductance), which inserts co-translationally (Facey et al., 2007) and the tail anchored membrane proteins TssL (SciP), DjlK, and Flk (Aschtgen et al., 2012; Pross et al., 2016; Peschke et al., 2018). In eukaryotes, the ER tail anchored membrane proteins with a high hydrophobic TM segment are inserted by the Get pathway while those proteins with low hydrophobic TM segment are inserted by the ER membrane protein complex (EMC) (Hegde and Keenan, 2021). Interestingly, Get1 and EMC3 of the Get complex and EMC, respectively, are YidC homologs found in the ER (Anghel et al., 2017; Chen and Dalbey, 2018; McDowell et al., 2021).

The common feature of substrates of the YidC only pathway is that they have a short translocated region (Hennon et al., 2015), suggesting that the YidC insertase has limited translocase activity. Indeed, if the polarity of the translocated domain exceeds a certain threshold by introduction of charged/polar residues, then YidC requires the assistance of the SecYEG complex, implying that YidC is incapable to translocate these substrates unaided (Soman et al., 2014; Hariharan et al., 2021). The switching from YidC-only pathway to the YidC/Sec pathway indicates that both the YidC and the SecYEG are surveying the polypeptide chain during the membrane insertion process. This is feasible by dynamic interaction of YidC with SecYEG (Sachelaru et al., 2017; Steudle et al., 2021) or, a certain portion of YidC is a part of the SecYEG/SecDFYajC/YidC holocomplex (Schulze et al., 2014).

**FIGURE 8:**
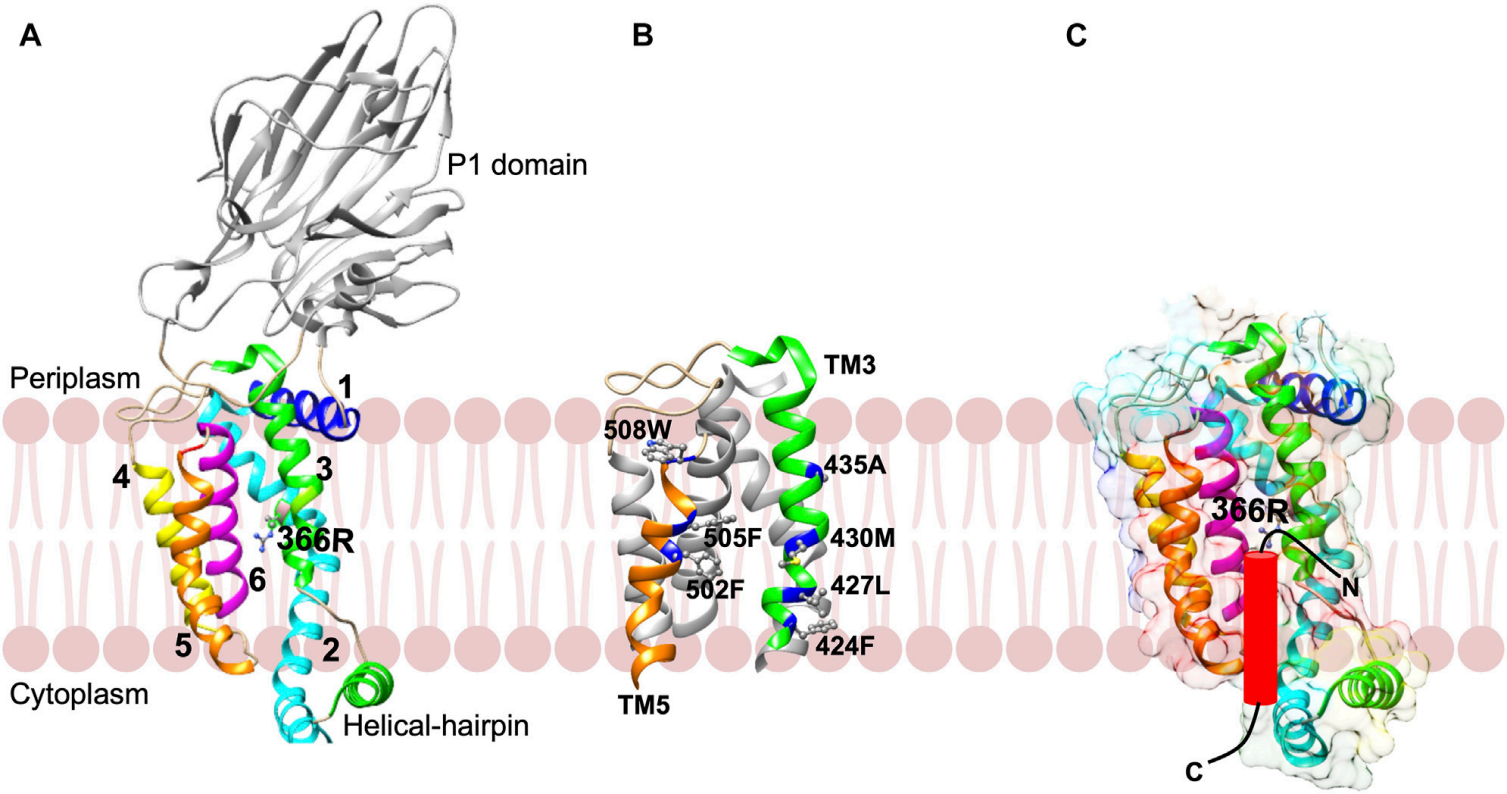
The YidC insertase [adapted from Kumazaki et al. (2014b) PDB: 3WVF]. **(A)** The *E. coli* YidC has a large periplasmic domain, a coiled cytoplasmic domain, and a conserved core region comprising of 5 TM helices (TMs 2–6) that form a hydrophilic groove open to the cytoplasm and lipid bilayer. The hydrophilic groove has a strictly conserved arginine that helps to keep the region hydrated. **(B)** A close-up view of the greasy slide (TM3 and TM5) that contacts the TM region of YidC substrates during insertion. The residues that contact the substrate TM segment (s) are indicated in dark blue. **(C)** During membrane insertion of the Pf3 coat, the TM segment moves up the greasy slide with the N-tail region captured transiently in the hydrophilic groove. The periplasmic domain of YidC is omitted in **(B,C)**.

Structural studies have shown that YidC is a monomer and contains a hydrophilic cavity within the 5 TM core region (Figure 8) (Kumazaki et al., 2014a; Kumazaki et al., 2014b). This aqueous groove with a conserved positively charged residue is open both to the cytoplasm and the lipid bilayer but closed from the periplasmic side. The existence of a hydrophilic groove located within the inner leaflet of the membrane was supported by *in vivo* solvent accessibility and MD simulation studies. The study also revealed that YidC shapes the membrane with significant membrane thinning around the protein (Chen et al., 2017). The presence of the hydrophilic groove in the membrane decreases the membrane crossing distance which would in turn reduce the energy cost of translocating a polypeptide chain. Wu and Rapoport (2021) have recently proposed that protein translocation through a locally thinned membrane is a new paradigm for lowering the energy cost for translocation.

Remarkably, the positively charged residue in the hydrophilic groove is essential for SpoIIIJ (YidC1) function in *B. subtilis*. It was proposed that the hydrophilic groove participates in an electrostatic step necessary to translocate the negatively charged N-terminal tail region of MifM across the membrane (Kumazaki et al., 2014a). However, the positive charge is not essential for the *E. coli* YidC (Chen et al., 2014). Rather, the positively charged residues plays a role in maintaining the hydrophilic microenvironment in the groove, which is necessary for the activity of YidC (Chen et al., 2022).

In addition to the hydrophilic groove, YidC has a cytoplasmic helical hairpin-like domain (Figure 8A) (Kumazaki et al., 2014a) which was predicted to be involved in the initial recruitment of the substrate. The arrangement of two antiparallel helices in the C1 region of EcYidC is rotated by 35° with respect to the core region, as compared to that in the BhYidC structure. Moreover, in both the structures the B factor for this region is high, demonstrating the flexibility of the C1 cytoplasmic loop region. Crosslinking studies of the essential C1 loop show contacts not only with the targeting proteins SRP and FtsY but also the Sec translocon (Geng et al., 2015; Petriman et al., 2018).

The mechanism of the substrate TM recognition by YidC is fascinating. Crosslinking studies have indicated that the TM3 of YidC contacts the TM domain of FtsQ, leader peptidase, subunit c of the F1FoATPase (Yu et al., 2008). Contacts are also observed with TM3 and TM5 of YidC to Pf3 coat and MscL (Klenner et al., 2008; Klenner and Kuhn, 2012). It has been proposed that the substrate enters the YidC groove between these TM3 and TM5 segments, which constitutes a greasy slide where the TM segment moves across the membrane (Figure 8B). Kedrov et al. (2016), performed cryo-EM studies on a YidC-ribosome Foc nascent chain complex where YidC was reconstituted in nanodiscs. The study revealed that the Foc nascent chain was in proximity to TM3 facing the lipid region.

He et al. (2020) elucidated the pathway employed by the single spanning Pf3 coat to provide insight into the YidC insertion mechanism of simple membrane proteins (Figure 8C). The tracking of the Pf3 coat protein through YidC was obtained by “freezing” each step of the insertion process by creating translational arrested intermediates of different sizes and investigating them by thiol crosslinking (He et al., 2020). The results divulged that the TM segment of Pf3 moves up the YidC greasy slide during membrane insertion. After the TM reached the top of the slide, the N-tail transiently enters the YidC hydrophilic groove. In the next step, the N-tail is released from the groove and translocated across the periplasmic leaflet of the membrane.

#### 2.3.3 TatC and TatC/SecYEG

The Tat substrates of *E. coli* include the five integral membrane proteins including HybO, FdnH, FdoH, HyaA and HybA (Hatzixanthis et al., 2003). The genes encode subunits of NiFe hydrogenase or formate dehydrogenase. They are encoded with a Tat signal peptide and possess a C-terminal TM segment that functions as a stop transfer domain. The recognition of these membrane proteins by the Tat complex is achieved by the interaction of the Tat signal peptide with the TatBC complex.

Although mechanistically different, surprisingly, in some bacteria, cooperation is observed between the Tat and the Sec pathway for the insertion and assembly of polytopic membrane proteins (Keller et al., 2012). The first evidence for this was from the analysis of the iron-sulfur membrane bound Rieske proteins from Gram-positive actinobacteria that has 3 TM segments. While the first 2 TM segments are inserted by the SecYEG translocase, the third TM segment, which is preceded by a Tat motif was inserted by the Tat system. To understand the mechanism of insertion further and to decipher the handover process from Sec to Tat, the *S. coelicolor* Rieske protein, Sco2149 was examined (Tooke et al., 2017). The authors observed that a moderate hydrophobicity of the TM3 segment and the presence of several C-terminal positively charged residues promote the release of the TM3 from the SecYEG apparatus. This further allows the Tat TM segment to engage with the Tat translocase and stimulate translocation across the membrane. Other examples of the dual participation by Sec/Tat machineries targeting membrane proteins include the five spanning membrane proteins *S. coelicolor* Molybdenum cofactor protein Sco3746, and the delta proteobacterium MLMS-1 FeS containing polyferredoxin. In these cases, the first 4 TM segments are inserted by the SecYEG complex, and the Tat system inserts the last TM segment and translocates the folded C-terminal domain. In each case, the fifth TM segment has moderate hydrophobicity and an amino terminal Tat RR-motif (Tooke et al., 2017).

### 2.4 Removal of the Signal Peptide and Degradation

The last step in the translocation pathway is the removal of the signal peptide. This allows the exported proteins to be released from the membrane so that they can continue their journey to the periplasm, outer membrane or to the extracellular medium. The removed signal peptides are degraded by enzymes having signal peptide hydrolase activity.

Signal peptidases cleave off the signal peptides and play crucial roles as endopeptidases with clear cut substrate specificities (Paetzel, 2019). Type I signal peptidase (SPase I; also known as leader peptidase) processes the majority of preproteins while Type II signal peptidases (SPase II; also known as lipoprotein signal peptidase) process lipoprotein precursors (Paetzel, 2014).

#### 2.4.1 Signal Peptidase I

The first signal peptidase to be characterized was *E. coli* signal peptidase I (SPase I). It was overproduced and purified to homogeneity (Wolfe et al., 1982). The purified protease in detergent was shown to cleave a wide variety of preproteins, including eukaryotic secretory preproteins (Watts et al., 1983). Similarly, the eukaryotic signal peptidase can cleave bacterial preproteins, demonstrating that the cleavage specificity is evolutionarily conserved (Watts et al., 1983). Subsequently, the *E. coli* protease enzyme was shown to be localized to the inner membrane with its catalytic domain facing the periplasmic space (Wolfe et al., 1983). Moreover, it was shown to be an essential enzyme for the bacteria (Date, 1983).

Signal peptidases are indispensable for the secretion process. Disruption of the signal peptide processing prevents the preproteins from arriving to their correct destination in the cell (Dalbey and Wickner, 1985). Under decreased SPase I activity in a depletion strain, the accumulated preproteins were translocated but were anchored to the inner membrane by the uncleaved signal peptide. Therefore, the function of signal peptidase is to release the exported protein from the membrane by removing the signal peptide so that they proceed to their destination. It is now established that SPase I processes the majority of non-lipoproteins that are exported by the Sec pathway or by the Tat pathway (Lüke et al., 2009).

**FIGURE 9:**
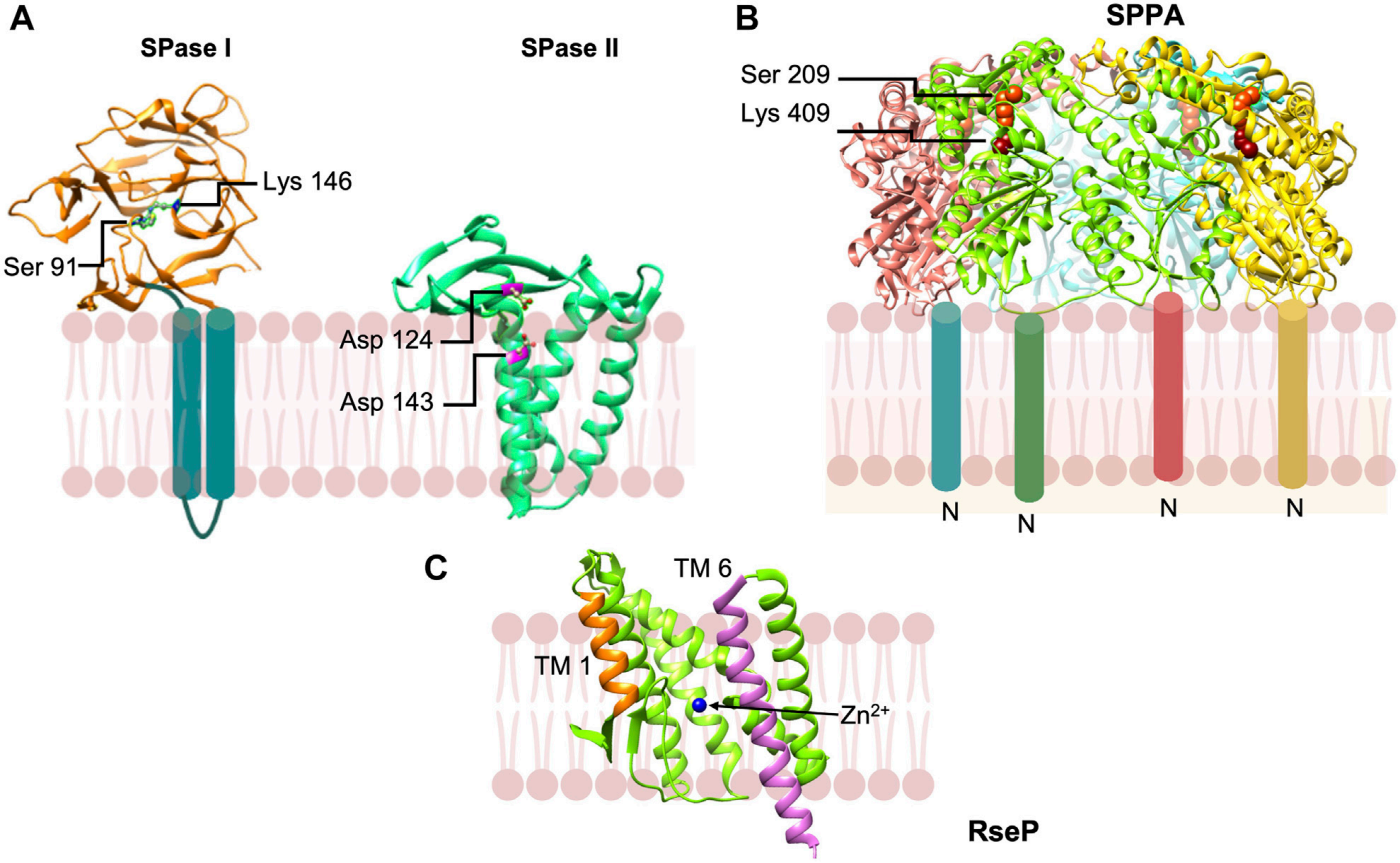
Peptidases involved in the removal of signal peptides and their degradation. **(A)** Signal peptidase 1 [adapted from Paetzel et al. (2004) PDB: 1T7D] is a novel Ser-Lys protease that cleaves the preprotein at the membrane surface on the periplasmic side. Signal peptidase 2 [adapted from Vogeley et al. (2016) PDB: 5DIR] is an aspartic acid protease that cleaves a diacyl glyceride modified preproteins within the plane of the membrane. **(B)** SppA [adapted from Kim et al. (2008) PDB: 3BF0] is a tetrameric protein that degrades signal peptides which are released from the membrane into the periplasmic space. SppA employs a Ser-Lys dyad and is anchored to the membrane by an amino terminal TM segment. **(C)** RseP signal peptide peptidase [from *Methanocaldococcus jannaschii* adapted from Feng et al. (2007) PDB: 3B4R] in open state. Both water molecules and peptide substrates reach the active site containing Zn^2+^ ion (blue) during its open state.

To understand how SPase cleaves and binds substrates at the active site, the structure of the *E. coli* signal peptidase periplasmic domain (Δ2-75) (Tschantz et al., 1995) was solved to high resolution of 1.9 Å (Paetzel et al., 1998) (Figure 9A). The catalytic serine (Ser 91) was covalently attached to the cleaved β-lactam inhibitor and the lysine 146 amino group was within hydrogen bond distance. This corroborates with the mutagenesis studies displaying the indispensable mechanism of active Ser and Lys dyad for catalysis (Sung and Dalbey, 1992; Tschantz et al., 1993; Paetzel et al., 1997) (Figure 9A) in contrast to the canonical Ser-His-Asp mechanism (Paetzel and Dalbey, 1997).

Intensive analysis of the structure of the active site region with the inhibitor (Paetzel et al., 1998) and a signal peptide modeled into the binding site of the apo enzyme (Paetzel et al., 2002) revealed the binding pockets at the S1 and S3 positions that account for the “-3 and -1” or “Ala-X-Ala” rule for processing based on conserved residues in preproteins (Heijne, 1983). The S1 pocket is quite small and the S3 pocket is slightly bigger, fitting well with the known substrate specificity. The -2, -4 and -5 residues are solvent exposed, consistent with almost any residue found at these positions. The substrate binding pocket was further supported by the structure of the *S. aureus* SPase I (SpsB) with a portion of the signal peptide and the early mature region sequence binding to the active site (Ting et al., 2016).

#### 2.4.2 Signal Peptidase II

As mentioned earlier, the substrate for signal peptidase II (SPase II) is a diacylglycerol modified prolipoprotein (Tokunaga et al., 1982). Following cleavage and further maturation, the bacterial lipoproteins possess a N-acyl diacylglycerylcysteine at the N-terminal end of the protein, which serves to anchor lipoproteins to the inner membrane or the outer membrane.

The gene for SPase II (lsp) was cloned by the Mizushima and the Wu labs independently (Tokunaga et al., 1983; Yamagata et al., 1983). The SPase protein spans the membrane four times with the protein ends facing the cytoplasm. The initial evidence of this peptidase as an aspartic protease was the fact that it was inhibited by pepstatin (Dev and Ray, 1984). Also, in *B. subtilis*, several aspartic acid residues located at the ends of the TM segments were shown to be important for activity (Tjalsma et al., 1999).

The structure of SPase II from *Pseudomonas aeruginosa* in complex with the inhibitor globomycin solved to 2.8 Å provided evidence that SPase II was an aspartic acid protease (Figure 9) (Vogeley et al., 2016). Along with mutagenesis studies, the work revealed that Asp 124 and Asp 143 comprise a catalytic dyad (Figure 9A). Interestingly, the aspartic residues are located within the predicted membrane region confirming that SPase catalyzes intramembrane proteolysis. These findings validate the fact that lipoprotein signal peptides typically have short hydrophobic regions. Caffrey and coworkers proposed a model for how SPase II binds the preprotein and carries out catalysis (Vogeley et al., 2016). The signal peptide helix of the preprotein enters the SPase II active region via TM segments 2 and 4, and then binds to the protein to position the preprotein lipobox residues analogous to the Leu-Ile-Ser tripeptide of the globomycin inhibitor. The signal peptide region immediately following the Leu residue in the lipobox is in an extended conformation with the Gly-Cys scissile bond positioned in proximity to the Asp catalytic dyad Asp124 and Asp143. The mature domain of the preprotein is located in the periplasmic region.

#### 2.4.3 Signal Peptide Degradation

The final step “in the life and death of signal peptides (see ref von Heijne, 1998)” is their degradation. Degradation of the signal peptides is important because in many cases they can be toxic to the cell or interfere with protein export (Wickner et al., 1987; Bolhuis et al., 1999). The first protease discovered to possess signal peptide hydrolase activity was SppA (or protease IV), which was shown to degrade the lipoprotein signal peptide (Hussain et al., 1982). SppA is an inner protein that forms a tetrameric structure (Hussain et al., 1982; Ichihara et al., 1984). The catalytic domain of SppA containing the active site Ser-Lys dyad (Figure 9B) (Kim et al., 2008; Wang et al., 2008) is found within the periplasmic region at a large distance from the membrane indicating that the signal peptide is released from the membrane prior to its degradation (Kim et al., 2008). Apparently, SppA would cleave a wide variety of signal peptides that are released into the periplasmic space. Other proteases such as oligopeptidase A would hydrolyze signal peptides that are released into the cytoplasm (Novak et al., 1986).

The bacterial RseP, like signal peptide peptidase in eukaryotes (Lyko et al., 1995; Brown and Goldstein, 1997; Weihofen et al., 2002), can catalyze cleavage of membrane spanning signal peptides (Saito et al., 2011). RseP is a site-2 protease that can cleave within TM segments of membrane proteins as well (Akiyama et al., 2004). It is a zinc metalloprotease (Figure 9C). The active site is in an aqueous environment close to the cytoplasmic surface of the membrane. It binds zinc and has an essential catalytic glutamic acid. Saito et al. (2011) showed that RseP is capable of degrading a number of signal peptides from a wide variety of preproteins such as OmpA, M13 procoat, LivJ, LivK, PhoA, TolC, SecM, suggesting that it significantly contributes to signal peptide degradation in *E. coli*. This group of proteases is fascinating since they catalyze proteolysis within the membrane.

## 3 Targeting the Signal Peptide Proteases as Antibiotic Target

Signal peptidase I and II are attractive antibacterial drug targets (Paetzel et al., 2000; Rao et al., 2014; Craney and Romesberg, 2015; El Arnaout and Soulimane, 2019; Upert et al., 2021). SPase I is conserved in bacterial pathogens and has a novel active site architecture (Ser-Lys dyad) that can be targeted. Its active site location on the periplasmic side of the membrane makes it readily accessible (Smith and Romesberg, 2012). SPase II presents as another target candidate as lipoprotein signal peptidases are absent in the eukaryotic organism. The reduced efficacy of the existing antibiotics and the emergence of drug-resistant bacterial pathogens has led to the urgent demand for new treatments. This warrants the study of novel antibacterial targets such as SPase I and SPase II.

### 3.1 SPase I as Antibiotic Target

**FIGURE 10:**
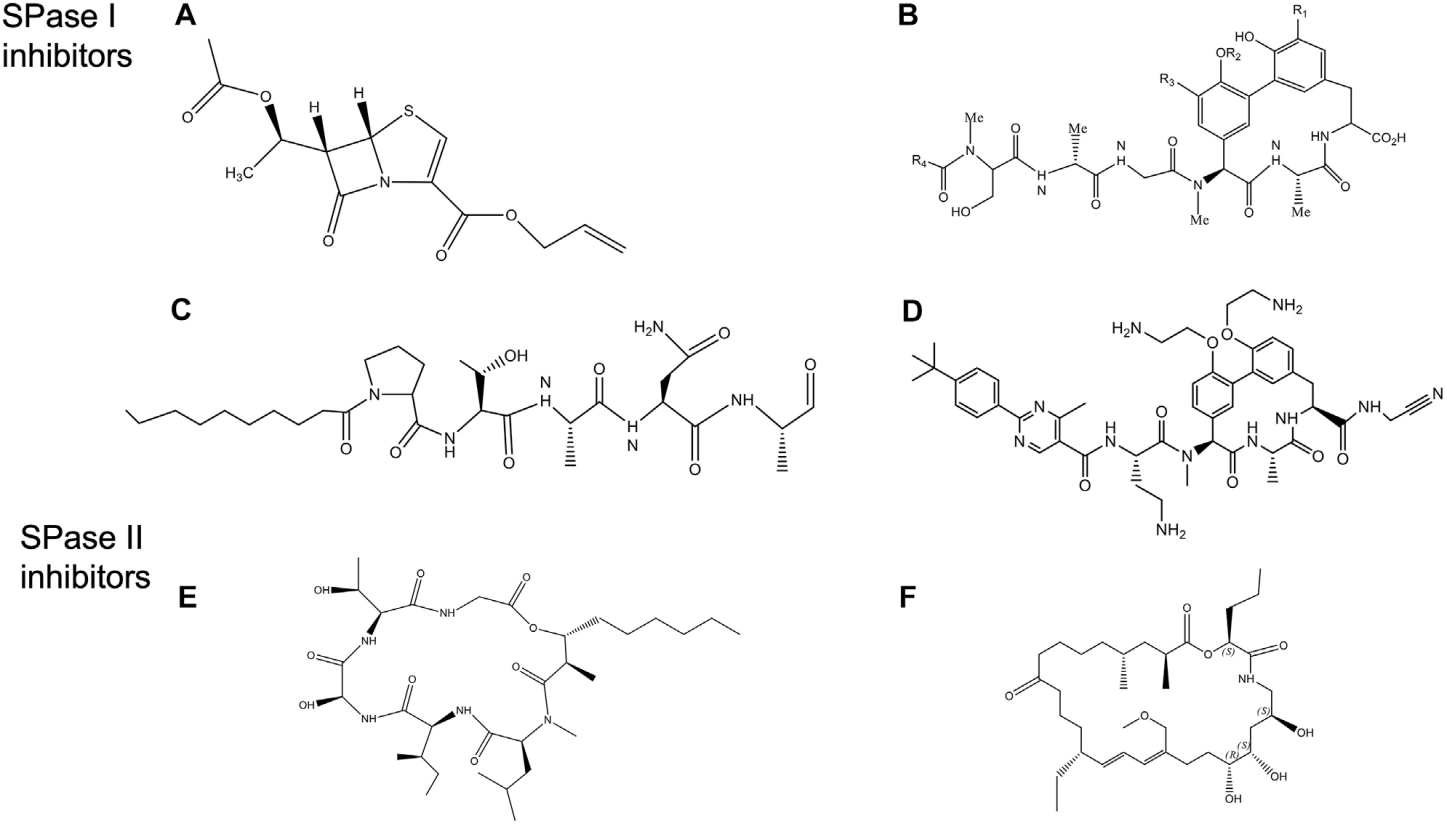
Structures of Signal peptidase inhibitors (Rao et al., 2014). **(A)** allyl (5S,6S)-6 [(R)-acetoxyethyl]-penem-3-carboxylate. **(B)** Arylomycin A. **(C)** Decanoyl PTANA aldehyde. **(D)** GO775, an optimized arylomycin. **(E)** Globomycin. **(F)** Myxovirescin.

Various companies have centered on SPase I as an antibacterial target including Smithkline Beecham pharmaceuticals (now GlaxoSmithKline), Merck, Eli Lilly and Genentech. Some of the first inhibitors were the β-lactams, including clavams, thioclavams, penem carboxylate C6 Substituted esters, and allyl (5S,6S)-6-[R)-acetoxyethyl]penem-3-carboxylate (Figure 10A) and (5S)-tricyclic penem (Black and Bruton, 1998). Some of the peptide inhibitors such as, α-ketoamide peptides and decanoyl-PTANA-aldehyde (Figure 10C) were effective (Bruton et al., 2003; Buzder-Lantos et al., 2009).

Another promising class of inhibitors are the natural products Krisynomycin and Arylomycins. They are produced in *Streptomyces* by the non-ribosomal peptide synthesis. The Arylomycin family includes Arylomycin A and B (Höltzel et al., 2002; Schimana et al., 2002), and Arylomycin C (a lipoglycopeptide) (Kulanthaivel et al., 2004) discovered in the beginning of the 21st century. More recently, Arylomycin D was discovered (see below). Arylomycin D and its derivative M131, as well as Krisynomycin displayed significant antibacterial activity against the methicillin resistant *S. aureus* (MRSA) (Therien et al., 2012; Tan et al., 2020). Romesberg and others have developed a total synthesis of Arylomycin A2 (Roberts et al., 2007), Arylomycins B2 (Dufour et al., 2010; Roberts et al., 2011), Arylomycin C (Liu et al., 2011) and members of the Arylomycin D class (Therien et al., 2012; Tan et al., 2020).

Noteworthy, Smith and Romesberg (2012) proposed that Arylomycins may represent a class of latent antibiotics whose activity can be masked by mutations in the SPase I protease which otherwise would have rendered them susceptible. While Arylomycins normally have a narrow spectrum of antibiotic activity (Figure 10B), these antibiotics have the potential to have a much broader spectrum antibiotic activity against both Gram-positive and Gram-negative bacteria. Romesberg and coworkers initially investigated *Staphylococcus epidermidis* that was sensitive to Arylomycin A and isolated resistant strains which had mutations in SPase I (Ser to Pro changes at position 29). Intriguingly, the analogous Pro mutations in SPases that occurred during evolution, accounted for the natural resistance that is observed in *E. coli*, *P. aeruginosa*, and *S. aureus.* Strikingly, they found that a wide variety of bacteria that lacked this Pro substitution were sensitive to Arylomycin.

To gain insight into how to improve the potency of Ayrlomycin the structure of the *E. coli* SPase-Arylomycin complex was solved (Paetzel et al., 2004). Paetzel and coworkers revealed that the carboxylate of Arylomycin was bound to the catalytic serine, and the penultimate alanine of the inhibitor was localized within the S3 pocket. The structure also shows that residue 84 of *E. coli* SPase that confers resistance to the antibiotic is positioned near the amino-terminal part of the Arylomycin lipopeptide. A proline at the 84 position prevents the donation of one potential hydrogen bond from the backbone amide group on the β-strand 1 of SPase I to the carbonyl oxygen of the fatty acid of arylomycin. The presence of this extra H bond interaction would presumably increase the affinity of arylomycin to the SPase I proteins, making the bacteria more susceptible to the antibiotic.

A potential breakthrough in the antibiotic field by scientists at Genentech was the production of G0775, which represents a new class of Gram-negative antibiotics, that targets SPase I (Figure 10D) (Smith et al., 2018). This optimized arylomycin had several modifications including a replacement for the natural aliphatic tail, modification of the phenol groups of the tripeptide ring, and the introduction of an electrophilic warhead at the C-terminal carboxylate. GO775 was 500 times more potent than the arylomycin A-C16 against *E. coli* and *K. pneumoniae* which was normally not inhibited by arylomycin. It also had a potent activity against other pathogens. Additionally, it was effective in treating mice that were infected with Gram-negative bacterial pathogens, without any toxic impact on the mammalian cells. Intriguingly, the electrophilic warhead that was expected to covalently modify the active site serine instead modified the catalytic base lysine, by a novel mechanism. High affinity interaction between the target protein and the inhibitor made G0775 extremely active against multidrug resistant bacteria.

### 3.2 SPase II as Antibiotic Target

SPase II has been an attractive antibiotic target since the natural products globomycin (Figure 10E) and myxovirescin (Figure 10F) have been shown to have antibacterial activity (Inukai et al., 1978a; Inukai et al., 1978b; Nakajima et al., 1978; Xiao et al., 2012). Globomycin is a cyclic depsipeptide produced in *Streptomyces*. Myxovirescin is a secondary metabolite with a 28-membered macrocyclic lactone that is made in *myxobacteria*. Inhibition of SPase II is lethal in all Gram-negative bacteria.

Quite surprisingly, the structures of the *S. aureus* SPase II in complex with either globomycin or myxovirescin (Olatunji et al., 2020) revealed that the mode of inhibition is similar despite the two antibiotics interacting mostly with the protein on opposite sides of the substrate binding pocket. While both inhibitors bind to the catalytic Asp dyad with the hydroxyl group wedging in between (the β-hydroxy group of serine residue of globomycin and a 6 OH group from myxovirescin), most of the remaining parts of the molecule were on opposite sides of the substrate binding region. The interaction of the OH with the aspartic acid behaved like a non-cleavable tetrahedral analog (Olatunji et al., 2020). The hydroxyl groups of the antibiotics inhibited the enzyme by targeting the catalytic dyad aspartic acid residues.

To identify new inhibitors of SPase II, a high throughput screen was performed (Kitamura et al., 2018) where 646,275 molecules were analyzed using a SPase II FRET substrate assay. To validate their assay, they showed globomycin inhibited SPase II with an IC50 of 1.2 nM. Myxovirescin had a comparable or even better IC50. After identifying the best molecules from this initial screening, further optimization of the compound by medicinal chemistry resulted in an inhibitor of IC50 of 99 nM. Although this was a potent SPase II inhibitor, it did not accrue antibacterial activity in *E. coli* unless it was used in combination with polymyxin B nonapeptide, which made the outer membrane more permeable to compounds.

More recently, the structure of Globomycin was optimized to improve its antibacterial activity against *E. coli* (Garland et al., 2020) and its permeability across the outer membrane. Taking advantage of the SPase-globomycin structure, modifications were made to alter the lipophilic side chains, the n-hexyl group, and the backbone to introduce a salt-bridge that interacted with the SPase II catalytic aspartic residues. Several compounds were obtained that had increased potency against several Gram-negative pathogens.

## 4 Summary and Outlook

In conclusion, the signal peptide plays a universal central role in protein export in the three kingdoms of life. It orchestrates the sorting of proteins from the cytosol to the membrane. After targeting the protein to the membrane by binding to the receptor, the signal peptide activates the translocase such that the preprotein can make its way to the other side of the membrane.

Activation of the SecYEG channel occurs by binding of the signal peptide to the lateral gate, leading to a conformational change in the channel. Thus, signal peptide binding unlocks the Sec channel for translocation. In case of the Tat translocation machinery, the signal peptide provides the signal for the assembly of an oligomeric Tat translocase capable of transporting fully folded protein substrates. The key to triggering this process is the twin arginines within the signal peptide that binds to the TatBC receptor which switches on the Tat assembly process.

The final step in the translocation process is the removal of the signal peptide and its degradation. The hydrophobic region of the signal peptide positions the cleavage site for proteolysis by signal peptidases. After having served its purpose of protein navigation, the signal peptide is degraded by signal peptide hydrolases.

Owing to the decisive role the signal peptidases play in protein transport process, they have been appreciated recently as novel targets for antibiotics. The proteins so inhibited are involved in an array of bacterial fundamental processes essential for growth and viability of the bacteria/pathogen. The recent studies present Arylomycin and its derivatives such as G0775 as promising candidates for translation into new medicine to treat multidrug resistant pathogens. These compounds and the next generation of synthetic analogs will hopefully prove to be successful antibiotics to combat bacterial infections.

Yet, much remains to be discovered in the protein targeting and export field even 50 years after the Signal Hypothesis was proposed by Günter Blobel. Snapshots of the machineries engaged in substrate translocation are expected to provide new mechanistic insight into the processes of translocation dynamics and orientations of polytopic membrane proteins. Protein export has entered an exciting chapter, and more is anticipated in the days to come.
